# Association of large core middle cerebral artery stroke and hemorrhagic transformation with hospitalization outcomes

**DOI:** 10.1038/s41598-024-60635-0

**Published:** 2024-05-01

**Authors:** Jack E. Pohlmann, Ivy So Yeon Kim, Benjamin Brush, Krishna M. Sambhu, Lucas Conti, Hanife Saglam, Katie Milos, Lillian Yu, Michael F. M. Cronin, Oluwafemi Balogun, Stefanos Chatzidakis, Yihan Zhang, Ludovic Trinquart, Qiuxi Huang, Stelios M. Smirnakis, Emelia J. Benjamin, Josée Dupuis, David M. Greer, Charlene J. Ong

**Affiliations:** 1 Department of Neurology, Boston Medical Center, 1 Boston Medical Center PI, Boston, MA 02118 USA; 2 Department of Epidemiology, School of Public Health, Boston University, 715 Albany St, Boston, MA 02118 USA; 3 Department of Neurology, NYU Langone Medical Center, 550 1st Ave, New York, NY 10016 USA; 4 Department of Neurology, Boston University School of Medicine, Chobanian and Avedisian School of Medicine, 85 E Concord St., Suite 1116, Boston, MA 02118 USA; 5 Department of Neurology, Brigham & Women’s Hospital, Harvard Medical School, 75 Francis St, Boston, MA 02115 USA; 6 Institute for Clinical Research and Health Policy Studies, Tufts Medical Center, 800 Washington St, Boston, MA 02111 USA; 7 Tufts Clinical and Translational Science Institute, Tufts University, 419 Boston, Ave, Medford, MA 02155 USA; 8 Department of Neurology, Jamaica Plain Veterans Administration Medical Center, 150 S Huntington Ave, Boston, MA 02130 USA; 9 Department of Cardiology, Boston Medical Center and Boston University Chobanian and Avedisian School of Medicine, 85 E Concord St, Boston, MA 02118 USA; 10 Department of Epidemiology, Biostatistics and Occupational Health, McGill University, 2001 McGill College, Montreal, QC Canada

**Keywords:** Stroke, Neurology, Risk factors

## Abstract

Historically, investigators have not differentiated between patients with and without hemorrhagic transformation (**HT**) in large core ischemic stroke at risk for life-threatening mass effect (**LTME**) from cerebral edema. Our objective was to determine whether LTME occurs faster in those with HT compared to those without. We conducted a two-center retrospective study of patients with ≥ 1/2 MCA territory infarct between 2006 and 2021. We tested the association of time-to-LTME and HT subtype (parenchymal, petechial) using Cox regression, controlling for age, mean arterial pressure, glucose, tissue plasminogen activator, mechanical thrombectomy, National Institute of Health Stroke Scale, antiplatelets, anticoagulation, temperature, and stroke side. Secondary and exploratory outcomes included mass effect-related death, all-cause death, disposition, and decompressive hemicraniectomy. Of 840 patients**,** 358 (42.6%) had no HT, 403 (48.0%) patients had petechial HT, and 79 (9.4%) patients had parenchymal HT. LTME occurred in 317 (37.7%) and 100 (11.9%) had mass effect-related deaths. Parenchymal (HR 8.24, 95% CI 5.46–12.42, p < 0.01) and petechial HT (HR 2.47, 95% CI 1.92–3.17, p < 0.01) were significantly associated with time-to-LTME and mass effect-related death. Understanding different risk factors and sequelae of mass effect with and without HT is critical for informed clinical decisions.

## Introduction

Life-threatening mass effect (**LTME**) is a critical condition in which space-occupying cerebral edema, with or without hemorrhagic transformation (**HT**) compresses crucial midline structures. LTME is observed in up to 40% of patients with non-revascularized Middle Cerebral Artery (**MCA**) ischemic stroke^1^. This condition, defined as the significant shift of brain structures due to increased intracranial pressure, leads to neurologic decline, disability, and up to an 80% mortality rate^2^ Despite these grave outcomes, decompressive hemicraniectomy (**DHC**) can significantly reduce mortality rates, though it is associated with considerable morbidity, necessitating careful patient selection.

The evolution of stroke treatment, particularly with the advent of mechanical thrombectomy, has significantly impacted management strategies and outcomes for acute ischemic stroke patients^3^. Yet, despite advancements, the differentiation between LTME resulting solely from cerebral edema versus LTME compounded by HT remains underexplored. This distinction is crucial, as the pathophysiological mechanisms underlying these conditions differ: cerebral edema typically results from fluid accumulation within brain tissues, leading to increased intracranial pressure, while HT involves bleeding into brain areas already damaged by ischemia, potentially exacerbating the mass effect and complicating clinical management. Moreover, it remains unclear to what extent HT associated with MT differs from HT in the absence of revascularization. These distinctions remain relevant even as MT eligibility has expanded in recent years, for those who remain ineligible.

Prior studies have delved into risk factors associated with LTME post-stroke^4–6^, but there remains a gap in literature specifically addressing the nuances between LTME secondary to cerebral edema and those instances compounded by HT. Differentiating between HT subtypes (parenchymal, petechial, none) is essential, as they have varying implications on treatment decisions and prognosis for this critically ill population. In light of this, our study aims to (1) describe the characteristics of HT subtypes in patients with large core MCA infarct and (2) examine whether parenchymal HT is associated with accelerated time to LTME and worse hospital outcomes compared to patients without HT.

## Methods

The study was approved to be conducted by the Mass General Brigham Institutional Review Board (2017P002564). All methods were performed in accordance with the relevant guidelines and regulations set forth by the Institutional Review Board. Due to the retrospective nature of the study, the Mass General Brigham Institutional Review Board waived the need of obtaining informed consent.

**Figure 1:**
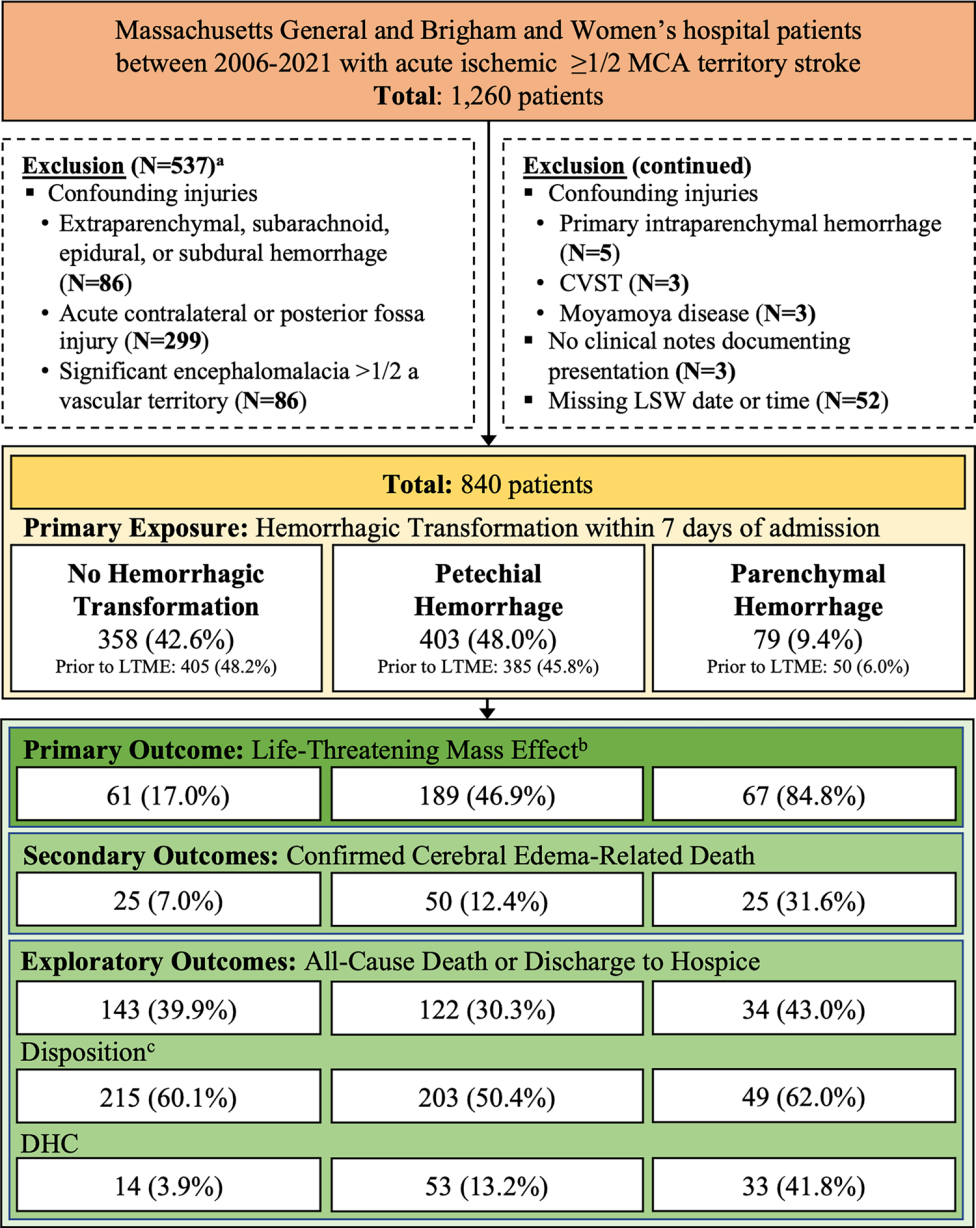
Study sample selection diagram. Patient flow sheet of inclusion and exclusion criteria. N (%) or Median [25th–75th]. *CVST* Cerebral Venous Sinus Thrombosis, *DHC* Decompressive Hemicraniectomy, *LSW* Last Seen Well, *MCA* Middle Cerebral Artery. ^a^Patients may meet multiple exclusion criteria. ^b^Hemorrhagic transformation classification occurs within 7 days of LSW. ^c^Long-term care, hospice, or death.

We conducted a two-center retrospective cohort study of adult patients with acute ischemic MCA stroke from the Brigham and Women’s and Massachusetts General Hospital admitted between 2006 and 2021 within 7 days of last seen well. We screened for acute MCA stroke using a previously described, highly sensitive Natural Language Processing algorithm^7^. All patients with possible large acute MCA stroke were confirmed to have a unilateral stroke size ≥ 1/2 of the MCA territory using direct visualization of radiology images on either CT or MR after acute interventions like tPA or MT by designated, trained, M.D members of the research team (SC, BB, CJO). Exclusion criteria are described in Fig. 1.

We collected clinical information from the electronic medical record using the Research Data Patient Registry and Enterprise Data Warehouse. Imaging related variables were collected by visual review by a trained team member (SC, BB, CJO) after establishing inter-rater-reliability (SC, BB, CJO). Detailed information on data collection and quality control for variables and outcomes of interest are included in the Supplementary Methods. We prepared this report according to Strengthening the Reporting of Observational Studies in Epidemiology reporting guidelines.

**Figure 2:**
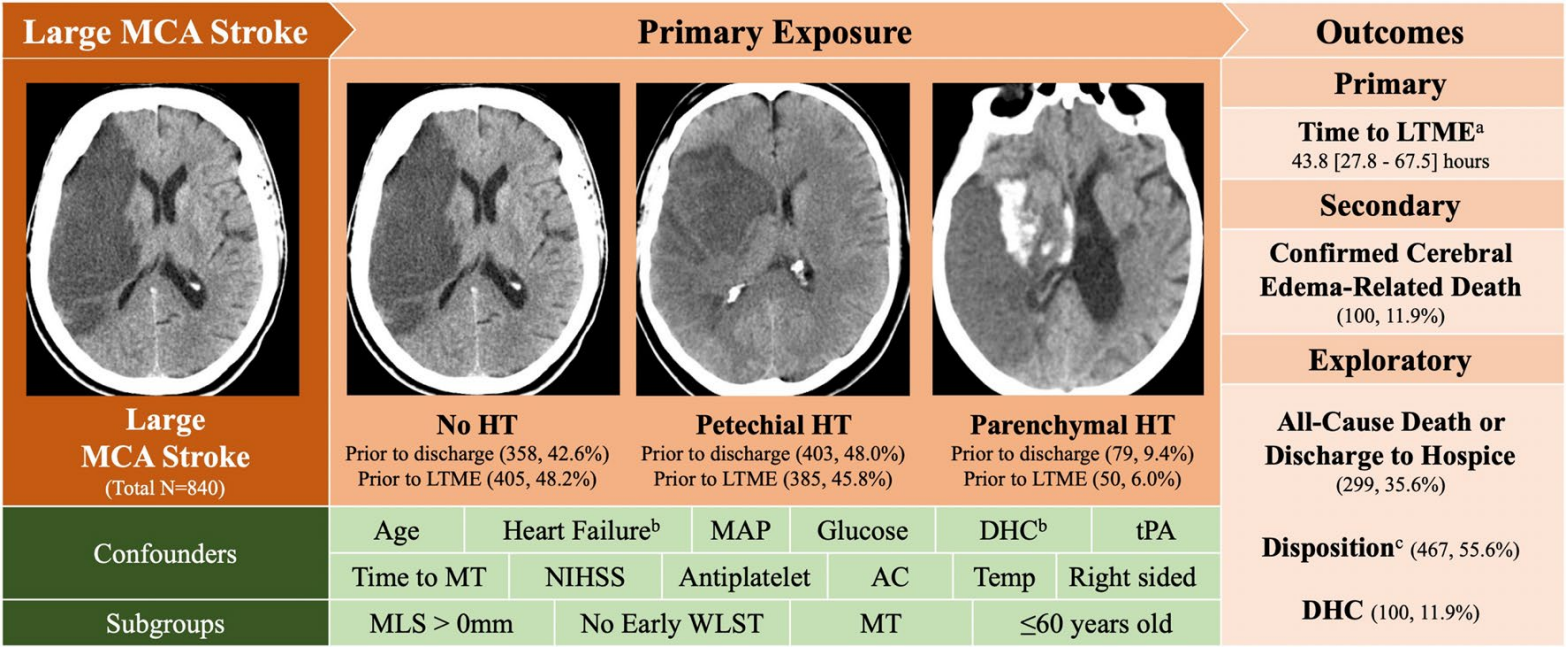
Study overview. Schematic of Exposures, Confounders and Subgroups for Primary, Secondary and Exploratory Outcomes. Representative images of no HT (42.6% of cohort), petechial HT (48% of cohort) and parenchymal HT (9.4% of cohort) shown below. N, % or Median [25th-75th]. *AC* Anticoagulation, *DHC* decompressive hemicraniectomy, *HT* hemorrhagic transformation, *LTME* life-threatening mass effect, *MAP* mean arterial blood pressure, *MCA* middle cerebral artery, *MLS* midline shift at septum pellucidum, *MT* mechanical thrombectomy, *NIHSS* national institute of health stroke score, *tPA* tissue plasminogen activator, *WLST* withdrawal of life sustaining therapy. ^a^Time to LTME (MLS ≥ 5 mm or DHC)-only hemorrhagic transformation prior to LTME was considered. Hemorrhagic transformation classification within 7 days. ^b^Heart failure and DHC were additional included covariates for the all-cause death and disposition models. ^c^Long-term Care, Hospice, or Death. Images W:70, L:30.

The primary outcome was time-to-LTME, defined as time from last seen well until imaging showed midline shift (**MLS**) measured at the level of the septum pellucidum of $\ge$ 5 mm or DHC (see Supplemental Methods for more detailed methodology). Under normal circumstances, the septum pellucidum is aligned centrally, indicating that the pressures in the two halves of the brain are balanced. MLS at the septum pellucidum indicates that there is some form of pressure differential across the two cerebral hemispheres. We chose 5 mm as an indicator of significant MLS as it has been used in other studies to predict malignant edema^8^. Also similar to other studies^6,9–11^, we used a composite clinical and radiographic outcome to identify life-threatening mass effect as it allows the capture of patients who clinically were felt to go on to have cerebral edema but otherwise would be censored by not having imaging confirmed substantive MLS. The secondary outcome was mass effect-related death, an outcome derived from chart review and team consensus, accounting for stroke size with associated MLS of at least 5 mm, temporality (either within traditional timelines of 2–5 days but screened up to two weeks for possible delayed edema cases), and lack of other possible causes. Further details are described in the supplementary analysis. We conducted exploratory analyses of all-cause death or discharge to hospice, disposition at discharge (long-term care, hospice, or death versus rehabilitation or home) and presence or absence of DHC only **(**Fig. 2**)**.

The primary exposure of HT subtype was defined as patients with no HT, petechial HT only, or parenchymal HT (with or without petechiae). Grading of petechial and parenchymal HT occurred through direct visualization according to the European Co-operative Acute Stroke Study (**ECASS II**) classification scheme^12^ on head computed tomography (**CT**) or Magnetic Resonance Imaging by a trained MD (SC). Multivariable model covariates were selected by reviewing the literature and included age, admission mean arterial pressure, admission glucose, intravenous tissue plasminogen activator (**tPA**), occurrence and time to mechanical thrombectomy (**MT**), National Institute of Health Stroke Scale (**NIHSS**), prior antiplatelet use, prior anticoagulation use, temperature, and right-sided stroke, which has been previously associated with a trend toward increased odds of LTME^13^. For the secondary outcomes of death and disposition, DHC and heart failure were additional model covariates^5,14–16^.

### Statistical analysis

We summarized patient characteristics at admission with means and standard deviations or frequencies and proportions. We used one-way analyses of variance or chi-square tests where appropriate to test for differences between the HT subtype groups.

For time-to-LTME, we used Cox regression. We considered HT subtype as a time-dependent covariate progressing from no HT to either petechial HT only or parenchymal HT. For LTME analysis, HT was only considered present if it occurred prior to or concurrent with LTME. LTME occurrence could be censored by death or discharge.

We used multivariable logistic regression for other outcomes, treating our primary exposure of HT subtype as a three-level variable with no HT as the reference group. We report odds ratios (**OR**) and p-values from Wald chi-square test on the coefficients. For DHC analysis, HT was only considered present if it occurred prior to DHC. For discharge outcomes, HT was considered present if it occurred within 7 days of stroke. We similarly report hazard ratios (**HR**) and p-values.

We performed sensitivity analyses of patients with at least some degree of MLS, early WLST within 24 h who would not have undergone subsequent imaging or care, patients who underwent MT, and patients under 60 years of age to mitigate potential biases conferred by stroke size, early withdrawal of life sustaining therapy (**WLST**), those who underwent MT, and age on outcome. We also performed a sensitivity analysis using HT classified only by CTs, as MR can have an increased sensitivity in detecting blood products.

We set the significance threshold at $\mathrm{\alpha }$=0.05/2 = 0.025 using a Bonferroni correction^17^ to address the multiple endpoint multiplicity issue and maintain the overall type I error at 0.05 across the association of parenchymal HT and time-to-LTME and mass effect-related death. Other analyses used $\mathrm{\alpha }$=0.05 and are hypothesis-generating only.

To handle missing values for structured variables (Supplementary Table 2), we employed multiple imputation by chained equations using the MICE package in R. For all analyses, we used RStudio Version 1.3.959 (*RStudio Team (2020). RStudio: Integrated Development for R. RStudio, PBC)* for statistical analyses. Further statistical details are available in Supplementary Methods.

## Results

Table 1 and Supplementary Table 7 describe admission characteristics of the 840 eligible patients (mean age 67.9, 48.2% female). Mean NIHSS was 17.2 ± 6.2, and mean Alberta Stroke Program Early CT Score (**ASPECTS**) was 4.4 ± 2.9. 41.3% of patients had strokes related to cardioembolism, 11.4% had strokes related to large artery atherosclerosis, 7.1% had strokes related to other determined etiology and 36.5% had stroke of undetermined etiology. Three-hundred and thirty-seven patients (40.1%) received intravenous tPA, and 142 (16.9%) received MT. In patients with MT, relevant door-to-needle and revascularization when available are included in Supplementary Table 1. Seventy patients (8.3%) were made comfort measures only within 24 h. Three-hundred and seventeen patients (37.7%) experienced LTME. One hundred (11.9%) received a DHC. One hundred patients had (11.9%) mass effect-related deaths. Two-hundred and forty-six patients (29.3%) of patients died of any cause or went to hospice by discharge. One hundred and sixty-eight patients (20.0%) went to long-term care, 342 (40.8%) went to rehabilitation, and 30 (3.6%) went home by discharge (Table 2).

**Table 1:** Baseline characteristics.

Variable	Total n = 840	Hemorrhagic transformation subtypes^a^			
		No hemorrhage	Petechial	Parenchymal	p-value
		n = 358 (42.6)	n = 403 (48.0)	n = 79 (9.4)	
Demographics					
Age	67.9 (15.5)	69.7 (15.9)	66.5 (15.3)	66.5 (13.7)	0.01
Female	405 (48.2)	195 (54.5)	182 (45.2)	28 (35.4)	< 0.01
Race					0.48
White	649 (77.3)	286 (79.9)	304 (75.4)	59 (74.7)	–
Black	53 (6.3)	21 (5.9)	27 (6.7)	5 (6.3)	–
Asian	33 (3.9)	10 (2.8)	17 (4.2)	6 (7.6)	–
Other^b^	105 (12.5)	41 (11.5)	55 (13.6)	9 (11.4)	–
Past medical history					
Hypertension	598 (71.2)	274 (76.5)	267 (66.3)	57 (72.2)	< 0.01
Atrial fibrillation	396 (47.1)	176 (49.2)	185 (45.9)	35 (44.3)	0.58
Heart failure	248 (29.5)	118 (33.0)	113 (28.0)	17 (21.5)	0.09
Prior stroke	93 (11.1)	54 (15.1)	33 (8.2)	6 (7.6)	< 0.01
Home medication					
Antiplatelets	246 (29.3)	112 (31.3)	111 (27.5)	23 (29.1)	0.53
Anticoagulants	98 (11.7)	40 (11.2)	46 (11.4)	12 (15.2)	0.59
statins	247 (29.4)	103 (28.8)	115 (28.5)	29 (36.7)	0.33
LSW to presentation (hours)	7.34 [3.93–17.8]	6.35 [3.87–16.2]	7.83 [4.02–21.6]	9.50 [5.25–19.2]	0.10
TOAST stroke subtype^c^					0.58
Large artery atherosclerosis	96 (11.4)	33 (9.2)	54 (13.4)	9 (11.4)	
Cardioembolic	347 (41.3)	153 (42.7)	159 (39.5)	35 (44.3)	
Small vessel occlusion	0 (0.0)	0 (0.0 )	0 (0.0)	0 (0.0)	
Other known cause of stroke	60 (7.1)	28 (7.8)	29 (7.2)	3 (3.8)	
Unknown cause of stroke	307 (36.5)	131 (36.6)	145 (36)	31 (39.2)	
Right sided stroke	435 (51.8)	173 (48.3)	217 (53.8)	45 (57.0)	0.20
Admission ASPECTS	4.4 (2.9)	4.6 (3.0)	4.3 (2.9)	4.2 (2.9)	0.25
NIHSS	17.2 (6.2)	17.2 (6.5)	17.3 (6.0)	17.2 (6.1)	0.97
Occlusion location	335 (39.9)	142 (39.7)	165 (40.9)	28 (35.4)	0.52
Internal carotid artery	151 (45.1)	64 (45.1)	75 (45.5)	12 (42.9)	–
M1	129 (38.5)	55 (38.7)	61 (37.0)	13 (46.4)	–
M2	51 (15.2)	23 (16.2)	25 (15.2)	3 (10.7)	–
M3 or M4	4 (1.2)	0 (0.0)	4 (2.4)	0 (0.0)	–
Acute intervention					
Tissue plasminogen activator	337 (40.1)	126 (35.2)	173 (42.9)	38 (48.1)	0.03
Mechanical thrombectomy	142 (16.9)	43 (12.0)	72 (17.9)	27 (34.2)	< 0.01
Admission labs					
HbA1c (mmol/mol)	6.2 (1.3)	6.1 (1.2)	6.4 (1.4)	6.4 (1.5)	0.04
Glucose (mg/dL)	150.8 (65.2)	145.7 (58.0)	155 (71.2)	152.5 (62.7)	0.14
White blood cell count (K/UL)	11.8 (7.6)	11.8 (5.4)	12.0 (9.6)	11.6 (4.1)	0.89
Creatinine (mg/DdL)	1.1 (0.8)	1.1 (0.8)	1.1 (0.7)	1.2 (1.1)	0.15
Sodium (mEq/L)	138.1 (3.6)	137.8 (3.7)	138.2 (3.6)	138.8 (3.1)	0.07
Admission vital signs					
Systolic blood pressure (mmHg)	149.2 (29.8)	149.1 (30.6)	149.1 (29.2)	150 (29.7)	0.97
Pulse (beats per minute)	82.7 (21.1)	82.8 (21.7)	83.9 (21.6)	78.1 (17.2)	0.36
Temperature (Fahrenheit)	97.9 (1.2)	97.6 (0.9)	98.0 (1.3)	98.2 (1.4)	0.07

Mean (SD), Median [25th–75th], or No. (%).

*ASPECTS* alberta stroke program early CT score, *LSW* last seen well, *MGB* mass general Brigham, *NIHSS* national institutes of health stroke scale, *toast*- trial of org 10,172 in acute stroke treatment.

^a^Hemorrhagic transformation classified by any HT within 7 days of LSW.

^b^Other Races, includes American Indian, Alaska Native, Native Hawaiian, Pacific Islander, Not Recorded, Not given or Unknown.

^c^Toast Classification: Of the 305 patients with stroke of Unknown Etiology, 42 had evidence of cardioembolic and large artery atherosclerosis or other known cause of stroke; Of 62 patients with other known cause of stroke, 24 had carotid dissection.

**Table 2:** Cohort Outcomes.

Variable	Total n = 840	Hemorrhagic transformation subtypes			p
		No hemorrhage	Petechial	Parenchymal	
		n = 358 (42.6)	n = 403 (48.0)	n = 79 (9.4)	
Mass effect					
Midline shift					
Midline shift > 0 mm	603 (71.8)	190 (53.1)	339 (84.1)	74 (93.7)	< 0.01
Midline shift ≥ 5 mm	295 (35.1)	54 (15.1)	178 (44.2)	63 (79.7)	< 0.01
Maximum midline shift (mm)	6.2 (4.7)	4.5 (2.9)	6.5 (4.2)	9.6 (4.7)	< 0.01
Treatment					
Osmotic therapy	282 (33.6)	81 (22.6)	157 (39.0)	44 (55.7)	< 0.01
Decompressive hemicraniectomy	100 (11.9)	14 (3.9)	53 (13.2)	33 (41.8)	< 0.01
WLST within 24 h					
DNR or DNI	187 (22.3)	102 (28.5)	68 (16.9)	17 (21.5)	< 0.01
Comfort measure only	70 (8.3)	43 (12.0)	18 (4.5)	9 (11.4)	< 0.01
Outcomes					
Time to LTME^a^ (hours)	43.8 [27.8–67.5]	46.7 [29.1–67.9]	49.6 [31.0–73.5]	31.8 [24.8–47.0]	< 0.01
Life threatening mass effect^b^	317 (37.7)	61 (17.0)	189 (46.9)	67 (84.8)	< 0.01
Confirmed mass effect-related death	100 (11.9)	25 (7.0)	50 (12.4)	25 (31.6)	< 0.01
All-cause death or hospice	299 (35.6)	143 (39.9)	122 (30.3)	34 (43.0)	< 0.01

Mean (SD), Median [25th–75th], or No. (%).

*DNI* do not intubate, *DNR* Do not resuscitate, *LTME* Life-threatening mass effect, *WLST* withdrawal of life sustaining therapy.

^a^Hemorrhagic transformation classified by any HT within 7 days of LSW.

^b^Of 317 patients who had LTME, 283 (89.3%) met definition by MLS $\ge$ 5, and 10.7% met definition through Decompressive Hemicraniectomy first.

Three-hundred and fifty-eight patients (42.6%) had no HT, 403 (48.0%) had petechial HT, and 79 (9.4%) had parenchymal HT within the first 7 days of admission. ECASS II subtype breakdown is included in the supplementary methods. Thirty-eight petechial HT (9.4%), and 29 (36.7%) parenchymal HT occurred after LTME, and were not included in the LTME survival analysis. Sixteen new petechial HT (4.0%), and 22 (27.8%) new parenchymal HT occurred after DHC, and were not included in the exploratory DHC analysis. (Supplementary Table 8).

Differences between non-HT, petechial HT, and parenchymal HT included age, female sex, hypertension, prior stroke, Hba1c, tPA, and MT (Table 1). Median time-to-petechial HT was 39.8 h and median time to parenchymal HT was 25.3 h. Petechial HT could and did occur prior to parenchymal HT in 29 of 79 patients. Median time-to-LTME occurred at 46.7 [25^th^-75^th^ range 29.1–67.9], 49.6 [31.0–73.5], and 31.8 [24.8–47.0] hours in patients with no, petechial, and parenchymal HT, respectively. Time-to-death did not appear substantially different between HT subtypes, though length of stay appeared longer in patients with hemorrhage (8 [4–13] v. 9 [6–16] v. 12 [6–19] days) (Supplementary Table 10). Univariable associations are included in Supplementary Table 4.

In the multivariable Cox model, parenchymal (HR 8.24, 95% CI 5.46–12.4, p < 0.01) and petechial (2.47 [1.92–3.17], p < 0.01) HT were significantly associated with time-to-LTME (Table 3). Parenchymal HT was associated with 9.25 [4.35–19.7] (p < 0.01) odds of mass effect-related death, and 4.39 [1.77–10.9] (p < 0.01) odds of DHC. Petechial HT was associated with two-fold odds of mass effect-related death (OR 2.18, CI [1.27–3.72], p < 0.01), but not DHC. There was no significant difference between HT subtype and all-cause death or discharge to hospice or discharge disposition, though there was a trend to significance for HT (p = 0.07) (Table 4).

**Table 3:** Time-to-LTME multivariable Cox proportional hazard regression (n = 840^a^).

Variables	Time to life-threatening mass effect^b^	
	HR (95 CI)	p
Parenchymal hemorrhage	8.24 (5.46–12.4)	< 0.01
Petechial hemorrhage	2.47 (1.92–3.17)	< 0.01

Abb.: LTME, Life-Threatening Mass Effect; CI, Confidence Interval; HR, Hazard Ratio.

Primary exposure: parenchymal HT; Secondary exposure: petechial HT.

^a^For the survival analyses, patients were classified into HT subtype based on images concurrent or prior to LTME. Four hundred and five had no HT, 385 had petechial only, and 50 had parenchymal HT.

^b^Midline shift ≥ 5 mm or decompressive hemicraniectomy; hemorrhagic transformation classification within 7 days.

**Table 4:** Multivariable logistic regression for secondary and exploratory outcomes (n = 840).

	Confirmed ME-related death		All-cause death or hospice		Disposition^a^		DHC^b^	
	OR (95 CI)	p	OR (95 CI)	p	OR (95 CI)	p	OR (95 CI)	p
Parenchymal	9.25 (4.35–19.7)	< 0.01	1.82 (0.96–3.45)	0.07	1.56 (0.86–2.84)	0.14	4.39 (1.77–10.9)	< 0.01
Petechial	2.18 (1.27–3.72)	< 0.01	0.75 (0.53–1.06)	0.10	0.77 (0.55–1.08)	0.13	1.55 (0.91–2.63)	0.10

Hemorrhagic transformation classification within 7 days.

*ME* mass effect, *CI* confidence interval, *DHC* decompressive hemicraniectomy, *OR* odds ratio.

^a^Long-term care, hospice, or death v. rehabilitation or home.

^b^For the DHC analysis, patients were classified into HT subtype based on images prior to of concurrent with DHC. Three-hundred and eighty had no HT, 402 had petechial only, and 57 had parenchymal HT.

We observed that parenchymal HT had significant HRs of LTME in all subgroups, and mass effect-related death (Supplementary Table 5). The association of petechial HT with secondary and exploratory outcomes in most subgroups were non-significant. Complete results are included in Supplementary Tables 4 and 5.

## Discussion

In patients with large MCA stroke, we found that parenchymal and petechial HT was associated with shorter time-to-LTME and increased risk of mass effect-related death. Our observation that patients with parenchymal or petechial HT progress more rapidly toward LTME may be in part due to the natural timeline of HT. Median time to symptomatic HT after tPA administration has been shown to be 18.25 h^18^, and may accelerate swelling from ischemia-related cerebral edema, which traditionally is thought to peak at 48–72 h after stroke onset^1^. We also found that patients with parenchymal and petechial HT had higher maximum MLS and were more likely to receive hyperosmolar therapy and DHC. While other studies have reported that the most severe ECASS II parenchymal HT subtype (PH-2) patients were associated with clinical deterioration and outcome^19,20^, it has not been previously established that parenchymal HT hastens the course of LTME and radiographic MLS. We found that petechial hemorrhage is also associated with a higher HR of LTME (p < 0.01) which in addition to our observation that petechial HT was associated with mass effect-related death (p < 0.01), may be contrary evidence to previous reports that petechial HT is not associated with poor outcome^19–21^. However, while we observed that petechial HT had a significant increased HR of LTME (p < 0.01), the longer LTME time compared to the no HT group is somewhat counterintuitive, as is the trend toward decreased all-cause death at discharge. We posit that patients with petechial HT had later petechial HT (median time-to-petechial HT 39.8 h v. 25.3 h) and subsequent LTME than their parenchymal counterparts, but was still associated with an increased HR of LTME once it occurred. Regarding the conflicting trend toward decreased all-cause death, we hypothesize that location and extent of petechial HT may impact its association with outcome, and may warrant further study to determine benign and malignant petechial phenotypes.

Parenchymal (p < 0.01) and petechial (p < 0.01) HT were significantly associated with mass effect-related death. This is consistent with the high rate of LTME (which is closely associated with mass effect-related death) in patients with parenchymal HT (84.8%) and petechial HT (46.9%) compared to none (17.0%). Our observation that HT was associated with mass effect-related death is consistent with the post hoc analysis of the ECASS finding of close to 50% mortality associated with PH-2 patients^19^. In our study, death or hospice by discharge was 43.0% for patients with any parenchymal HT, and 58.1% in patients with PH-2. Parenchymal HT was also associated with increased odds of receiving DHC in all subgroups except patients who received MT (p = 0.08).

Neither patients with parenchymal nor petechial HT were significantly more likely to experience all-cause death or hospice than those with no HT, though parenchymal HT trended toward significance. While the rate of all cause-death was slightly lower in patients with petechial HT, it was nonsignificant. The lack of a statistical association may be due to the high mortality in all groups given the severity of ischemic stroke; death due to causes outside of mass effect may occur similarly between the three groups. It is also possible that DHC may have resulted in decreasing the mortality relative to the no HT group, as there was a marked difference in the percent of patients who received DHC (42 v. 4% of parenchymal HT v. no HT). This potential rationale would be in line with prior studies showing DHC can decrease death from 80 to 20%^2,22–25^. We posit that DHC may have occurred more frequently because parenchymal HT tends to occur within the guideline-recommended < 48 h from stroke onset and appeared to occur in slightly younger patients (mean of 66.5 v. 69.7 years of age). Of our 246 deaths by discharge that 100 (40.7% of deaths) were confirmed due to mass effect, 90 (36.6%) were indeterminate, and 55 (22.4%) were confirmed from other causes. Disposition was also not significantly different in patients with parenchymal or petechial HT.

Similar to other studies, HT appeared to be associated with male sex, MT, temperature, stroke side, Asian race (parenchymal), HbA1c (petechial), and age (petechial) in our cohor^26–31^. HT is a known risk factors of tPA, in which symptomatic HT occurs in 2–7% of patients^16^.

Increased HT risk after MT is thought to be secondary to reperfusion injury of damaged tissue^15^. It is notable that in our cohort there are more parenchymal HT patients with MT than petechial or no HT patients (34.2 v. 17.9 v. 12%). It is possible that over the course of the study period technical improvements in MT procedures and patient selection may result in less HT in a “MT rich” era, after multiple studies demonstrating its benefit^3^. Delays in MT, either due to patient presentation time or door-to-needle time also raises concern for increased HT risk. In our population, we did not note substantive differences between door to needle time—in fact there was a slight increase in door to needle time for the no HT population (7.4 v. 6.2 v. 5.3 h). We expect that because the period of our study was largely before the adoption of MT policies that included either late-presenter and/or larger core infarcts^32–34^ that small differences between MT times did not substantively impact HT occurrence.

Regarding other associations we observed, acute hyperglycemia is associated with the development of cerebral edema, which may lead to worsened vascular compression and HT predilection^6^. HbA1c as a marker of chronic hyperglycemia may be reflective of diabetic microangiopathy associated with increased vulnerability to hemorrhage^35^. Hyperthermia has been shown to be associated with blood–brain barrier disruption in animal models^36^, and studies show that impaired cardiovascular regulation due to right-sided stroke^37,38^ may contribute to overall increased edema, injury, and potentially HT formation^31^.

Unlike other studies which found that amount of hypodensity was associated with HT^26^, we did not find a significant difference in stroke volume (as approximated by baseline ASPECTS score) and parenchymal HT. The observed differences between our study’s findings and others could partially be attributed to our predefined large-volume stroke population of interest ($\ge$ 1/2 MCA territory) as well as our three-group differentiation of HT instead of the traditional HT v. non-HT. Stroke size as a factor for HT might not be as important when all sizes of the stroke in the groups are more than half the MCA territory. Other notable findings include that patients with parenchymal HT received DHC later than patients without HT (median time-to-DHC of 40.4 v 34.9 h, respectively), which may suggest that the occurrence of HT prompts life-saving heroic measures when it occurs even outside of the customary 48 h window. Furthermore, less than a third (31.5%) of MCA stroke patients were 60 years or younger. This is a clinically meaningful observation, given that standard guidelines provide strong recommendations for preemptive DHC in patients $\le$ 60 only^1,39,40^. Given that more than two-thirds of patients are older than 60, and therefore do not have strong recommendations for practice, it may be worth considering therapeutic trials that focus specifically on an older cohort, contrary to most existing studies.

Prior well-designed clinical trials have led to post hoc investigations of HT and outcome, but only in broad cohorts with any stroke size after receiving medical or mechanical intervention^15,16^. The association of HT to outcomes in patients at risk from LTME due to large core stroke, especially after more regular use of acute stroke interventions including tPA and MT is less well characterized. Distinguishing the course of patients with different HT subtypes in large stroke is clinically relevant because they may have a different timeline for their peak MLS, respond differently to clinical interventions, and ultimately go on to have different outcomes. This is especially important in the era in which there is increasing evidence supporting acute interventions in patients with large core infarct^33,41^.

We recognize several important limitations of our study. It is a retrospective, observational study and therefore residual confounding cannot be excluded. Our cohort came from two hospital systems from a single region in which the majority of the population is White and generalizability is uncertain. We also acknowledge the limitations of our composite outcome, MLS $\ge$ 5 mm or DHC. Age and atrophy and anatomy can influence how devastating a given amount of midline shift can be. However, we used 5 mm as it has previously been used as indicator of severe cerebral edema because is highly associated with mortality and need for DHC^4,6,42^. We also adjusted for age as a covariate, though we were not able to for atrophy or anatomy. While DHC is an important marker for clinical judgement of life-threatening mass effect, we recognize that there is heterogeneity in its timing between clinical providers, centers, and over time. Despite its limitations, our composite outcome enables a feasible way to capture of timing of a devastating complication in a large dataset and to determine whether different variables like HT have different courses and outcomes. To amass a sufficient sample size, our retrospective study included patients over a long time-period in which there were developments that could alter clinical behavior, including regular use of MT. We found 33.6% of patients prior to 2015 had LTME, versus 46.5% thereafter. We posit that along with increasing use of MT, there may have been less early WLST. While the methods of intervention, particularly concerning MT, have evolved significantly, we believe that data on patients from before the widespread adoption of MT remains important and applicable to current practices. This is particularly true as not all patients, especially those from lower socio-economic backgrounds, are able to receive MT promptly or before major injuries occur, thus limiting the full benefits of this treatment.

HT was determined by imaging review, and petechial HT can be better seen on magnetic resonance imaging, which may have led to differences in how petechial HT was classified in patients with CTs only. Moreover, some studies had available susceptibility weighted imaging whereas others only had T2*-weighted gradient-echo sequence, which may have led to some heterogeneity in how small petechial hemorrhage was detected. However, our results were consistent when using only CTs to classify hemorrhages (Supplementary Table 4). Not all patients had an NIHSS. To mitigate this, we created inferred NIHSS from physical exams, possibly leading to artificially lower NIHSS (see Supplementary Methods). While we did not have precise stroke/hemorrhage volume, Demestere’s group reported that patients with ASPECTS < 7 reliably identified core volumes of > 70 cc^43^, suggesting that our mean ASPECTS score of 4.4 ± 2.9 is well within traditionally defined large MCA stroke volumes used in other studies^44^. Moreover, a random sampling of 30 patients using an ABC/2 method^45^ demonstrated that median volume was 122 cc^3^ (Supplementary Methods). There were no scheduled time points of imaging, however, the median frequency of imaging data within the first 48 and 72 h was 8.16 [3.97–16.4] and 10.6 [4.98–19.9] hours respectively, suggesting that most patients were closely observed to detect our endpoint (Supplementary Methods).

We did not have data on functional outcome either on discharge or 3 months, or neurologic deterioration. Early WLST within 24 h occurred in 22.3% of patients. It is important to acknowledge the bias conferred by WLST may confound our results, obfuscating the risk factors that may lead to unobserved MLS, and potentially misclassifying patients who may have otherwise survived their injury and subsequent mass effect. Finally, we were limited in exploring the association of delays to recanalization due to small sample sizes and because we did not have consistent available data for our whole cohort.

Despite these limitations, the strengths of our study are notable. We meticulously gathered and ascertained a comprehensive set of clinical, laboratory, and radiographic data from a broad cohort of patients across various hospital centers, ensuring a robust dataset. This extensive collection allowed us to classify cerebral edema phenotypes based on hemorrhagic transformation (HT) subtypes, providing novel insights into the distinct risk factors and outcomes associated with each HT category.

We found that patients with large MCA stroke who experience parenchymal HT are more likely to rapidly progress to LTME, and have high mortality, despite a higher rate of DHC. We also observed that patients with petechial HT were also at higher risk of quicker progression and mass effect related death than patients with no HT. Understanding the different clinical courses of HT subtypes in patients with large MCA stroke is important for clinical decision making and prognostication.

### Supplementary Information

Supplementary Information.

## Data Availability

Deidentified data can be made available upon request. For access, please email the lead and corresponding authors (JEP, CJO) with an explanation of your intended use. We will review your request, and if approved, provide data within 7 days. We reserve the right to withhold identifiable information such as external patient identifiers, dates of birth, and patient notes. All dates may be consistently shifted back in time up to 364 days so they do not reflect the actual dates, but preserve the intervals between them.
