# Supplementary material for: Association of large core middle cerebral artery stroke and hemorrhagic transformation with hospitalization outcomes
Source: Sci Rep. 2024 May 1;14:10008. doi: 10.1038/s41598-024-60635-0 (PMC11063151; doi:10.1038/s41598-024-60635-0)
Supplement: Supplementary file 1 — Supplementary Information. [file 41598_2024_60635_MOESM1_ESM.pdf]

Supplementary Methods: Association of large core middle cerebral artery stroke and hemorrhagic transformation with hospitalization outcomes.

Jack E. Pohlmann, MS[1,2], Ivy So Yeon Kim, MPH[1], Benjamin Brush, MD[3], Krishna M. Sambhu, BA[4], Lucas Conti, BA[4], Hanife Saglam, MD[5], Katie Milos[1], Lillian Yu[1], Michael Cronin, BS[4], Oluwafemi Balogun, MD, MPH[1], Stefanos Chatzidakis, MD[5], Yihan Zhang, MS[1], Ludovic Trinquart, PhD[2,6,7], Qiuxi Huang, PhDc[2], Stelios M. Smirnakis, MD, PhD[5,8], Emelia J. Benjamin, MD, ScM[2,9], Josée Dupuis, PhD[2,10], David M. Greer, MD, MA[1,4], Charlene J. Ong, MD, MPHS*[1,4,5]

[1]Department of Neurology, Boston Medical Center, 1 Boston Medical Center PI, Boston, MA 02118

[2]Department of Epidemiology, Boston University School of Public Health, 715 Albany St, Boston, MA 02118

[3]Department of Neurology, NYU Langone Medical Center, 550 1st Ave, New York, NY 10016

[4]Boston University School of Medicine, Chobanian and Avedisian School of Medicine, 85 E Concord St, Boston, MA 02118

[5]Department of Neurology, Brigham & Women's Hospital, Harvard Medical School, 75 Francis St, Boston, MA 02115

[6]Institute for Clinical Research and Health Policy Studies, Tufts Medical Center, 800 Washington St, Boston, MA 02111

[7]Tufts Clinical and Translational Science Institute, Tufts University, 419 Boston, Ave, Medford, MA 02155

[8]Department of Neurology, Jamaica Plain Veterans Administration Medical Center, 150 S Huntington Ave, Boston, MA 02130

[9]Department of Cardiology, Boston Medical Center and Boston University Chobanian and Avedisian School of Medicine, 85 E Concord St, Boston, MA 02118

[10]Department of Epidemiology, Biostatistics and Occupational Health, McGill University, 2001 McGill College, Montreal, QC, Canada

***Corresponding Author Information**

Charlene J. Ong, MD, MPHS

Assistant Professor, Dept. of Neurology

Boston University Chobanian & Avedisian School of Medicine

85 E Concord St., Suite 1116

Boston, MA 02118

Phone: 617-638-8647

Email: cjong@bu.edu

Supplementary Methods

These methods have been provided by the authors to give readers additional information about their methods and work.

Table of Contents

DATA COLLECTION **4**

ASCERTAINMENT/PROCESSING/CLEANING 4

EXPOSURES AND OUTCOMES **10**

ANALYSIS **12**

SUPPLEMENTARY TABLES **14**

DATA COLLECTION

Demographic and outcomes data were extracted from the electronic medical record at Massachusetts General Brigham using the Research Patient Data Registry (**RPDR**) for structured data, and radiology reports and clinical notes for unstructured data. A second researcher verified all data to ensure accuracy.

Ascertainment/Processing/Cleaning

We include the following description of relevant decision rules regarding ascertainment and cleaning of retrospective data.

The **presentation of a patient to the hospital** was approximated by the date and time of their first radiology report.

Age was derived by using the date of encounter minus the date of birth, rounding down to the year. **Sex**, **race**, and **ethnicity** were obtained through structured RPDR.

Past medical history used the following diagnosis codes on admission (except for ischemic stroke) or prior to admission atrial fibrillation (**Afib**), hypertension, and stroke.

- *Afib*[1] (ICD-9: [427.31, 427.32, 427.33], ICD-10: [I48.91, I48.1, I48.0, I48.92, I48.3, I48.4, I48.9])
- *Hypertension*[2] (Any of the following validated diagnosis codes present on admission or prior to admission of interest in RPDR: ICD-9: [401.x, 402.x, 403.x, 404.x, 405.x], ICD-10: [I10.x, I11.x, I12.x, I13.x, I15.x])
- *Heart Failure*[3] (Any of the following validated diagnosis codes present on admission or prior to admission of interest in RPDR: ICD-9: [428, 398.91, 402.01, 402.11, 402.91, 404.01, 404.03, 404.11, 404.13, 404.91, 404.93], ICD-10: [I50.1 to I50.9, I11.0, I13.0, I13.2])
- *Stroke*[4] (Any of the following diagnosis codes present prior to admission of interest in RPDR: ICD-9: [433.x1, 434.x1, 435.x, 436, 362.3], ICD-10: [I63.x, I64.x, H34.1, G45.x]).

Procedures/Treatments included:

- *IV tissue-Plasminogen Activator* (***tPA***) was obtained through RPDR and verified using clinical patient note reviews: Keywords: ["Mechanical", "Thrombectomy", "Solitaire", "Endovascular", "Recanal", "Catheter", "Stent", "Pass", "Penumbra", "Clot retrieval", "MERC/MERS", "IA therapy", "IA intervention", "Intraarterial", "Embolectomy"]. For instances in which RPDR and clinical patient notes were in conflict, (for example RPDR tPA was not recorded but clinical notes indicated that tPA was administered at an outside hospital) clinical patient notes were favored.)
- *Mechanical Thrombectomy* was obtained through RPDR using procedural codes and clinical patient note reviews. Reviewed from clinical notes; keywords including ["mechanical", "thrombectomy", "solitaire", "endovascular", "recanal", "catheter", "stent", "pass", "penumbra", "clot retrieval", "MERC", "MERS", "IA therapy", "IA intervention", "intraarterial", "embolectomy"]. For instances in which RPDR and clinical patient notes were in conflict, clinical patient notes were favored.

Mechanical Thrombectomy Time was determined by clinical note review. Types of reports including: "H&P," "Consults", "Final reports of FL Carotid Stenting", "FL stroke Intervention", "FL embolization", "FL Catheter Placement", "IR Acute Stroke Intervention." To determine MTDT we reviewed all available date times from the medical record including anesthesia start time, puncture time, recanalization time (if reperfused) and procedure note time. Not all cases had consistent reporting of these values. Therefore, 2015 and earlier, when documentation times were not as standard, the procedure note was used as the time of MTdt. Post 2015 when available, puncture time and/or anesthesia start time was used for MTdt. Finally, to obtain approximate MTDT time points in the remaining cases, either recanalization (N=1) or imaging surrounding the procedure was used (N=5) (**Supplementary Table 1**).

TICI (Thrombolysis in Cerebral Infarction) Score was obtained via clinical procedural notes. During instances in which two TICI scores were recorded, we chose the highest score. In instances in which TICI score was not explicitly recorded but a description regarding reperfusion was recorded (example: "full successful recanalization"), notes were reviewed by the clinical team a TICI score was assigned: no MT, no TICI if "Groin was punctured but no attempt"; TICI 0 if "Unsuccessful," "failed"; TICI 1 if "Minimal perfusion"; TICI 2C or 3 if "Full successful recanalization," "complete recanalization"; MT and TICI unknown if no information available.

*Decompressive Hemicraniectomy (**DHC**)* was obtained through RPDR using procedural codes (CPT 61322, CPT 61323, CPT 61304) radiology reports, and clinical note review. Any discrepancies were reviewed by the clinical team to ensure accuracy.

Surgery Time was determined by clinical note review (anesthesia start, surgery start). For clinical note that did not have surgery time listed, the date time of the first radiology report showing surgical decompression was used.

Osmotic Therapies (Dichotomously recorded if any of the following three osmotic therapies were administered over the course of admission. Other preparations of osmotic medications besides those listed below are not historically used at these institutions.

- *Hypertonic Saline, 3%* (Obtained administration date data through structured RPDR Codes based on Cost Center local medication designation string term search: "905698." Longitudinal dates and times were subsequently recorded for patients 2015-2021 from pharmacy records at Brigham & Women's Hospital (BWH) and Massachusetts General Hospital (MGH)
- *Hypertonic Saline, 23.4%* (Obtained presence of administration date data through structured RPDR. Codes based on string term search: 914402. Longitudinal dates and times were subsequently recorded for patients 2015-2021 from pharmacy records at BWH and MGH
- *Mannitol* (Obtained presence of administration date data through structured RPDR. Codes based on RegEx term search: 942788, J2150, 941586, 906010, 940665, 447, 941174 or 4749. Longitudinal dates and times were recorded for patients 2015-2021 from pharmacy records at BWH and MGH

Stroke Characteristics

*Last seen well (**LSW**)* date times were identified via clinical notes. LSW was considered the last time before a patient had a substantial deficit as inferred by National Institutes of Health Stroke Score (**NIHSS**) (symptoms of numbness, tingling, or other symptoms that

would result in NIHSS < 3 were not taken as the time of LSW). LSW without exact times or only reference to time were labeled as “inferred”. If LSW was prior to an in-hospital surgery, the surgery time was identified. If no surgery start time was identified, surgery was assumed to be at 7:00AM. If LSW referred to “last night before bed,” with no exact time, then LSW was assumed to be 10:00 PM. All last seen well data underwent supervisor review (CJO).

NIHSS was extracted from history and/or physical exam. In cases in which multiple NIHSS were reported, the last one (after any procedures took place) was used. Documented NIHSS via clinical notes. NIHSS after any thrombolytic procedure at BWH or MGH. Thrombolytic procedure included Therapeutic IV-tPA, Intra-arterial tPA, Mechanical thrombectomy with IA tPA, *and* Mechanical thrombectomy without IA tPA. If post-procedure NIHSS at BWH or MGH was not documented, then NIHSS upon arrival at BWH or MGH using the arrival exam was used. If the documented arrival and post-procedure NIHSS were not available, then NIHSS at the outside hospital (OSH) before the patient was transferred to BWH or MGH was used. If any thrombolytic agent was given OSH, then post-administration NIHSS was used. If post-procedure NIHSS at OSH was not available, then the arrival NIHSS at OSH was used. *Inferred NIHSS (*Previous studies have shown that the NIHSS can be estimated from the review of medical records with a high degree of reliability and validity.[5] For the cases where admission NIHSS was not explicitly stated in the patient’s admission record, it was inferred based on the available history and physical exam by a rater who was previously trained and certified in the administration of the NIHSS. Items on the scale were only scored if they were written in the HPI or physical exam portion of the admission note. Patient’s intubation status was assessed from recorded vital signs. Level of consciousness (scale item 1), best language (9), and dysarthria (10) were scored based on the HPI and mental status exam. Cranial nerve examination was used to score best gaze (item 2), visual fields (3), and facial palsy (4). The motor exam was used for motor arm (5) and motor leg (6). Limb ataxia (7) and sensory (8) were acquired from the ataxia and sensory sections of the neurological exam. Often, motor and sensory scores was inferred based on the patient’s reaction to noxious stimuli. For example, if the patient triple flexed to noxious stimuli, sensation was scored as "normal" and motor as “some effort against gravity” on the scale. Extinction and inattention (11) were scored based on mental status, cranial nerve, and sensory examinations. In cases where sections of the neurological exam were either incomplete or absent, those items were not scored on the scale.[5] We made the table of inferred NIHSS available in our Missing Value table (**Supplementary Table 2**)

Occlusion location (All CT Angiograms (**CTA**) were reviewed and classified as occlusion based on radiology reports. The most proximal vessel involved (Internal Carotid Artery, M1, M2, M3/M4) was used for the occlusion location. In instances in which reports described vessel occlusion but did not explicitly name vessel occluded, M1 was assigned if occlusion was described to begin prior to bifurcation/trifurcation.

*Anterior Cerebral Artery (**ACA**)* territory infarct was noted if hypodensity was present in the ACA territory (either A1 or A2 distributions).

Subtype of Acute Ischemic Stroke classification was conducted based on the original paper by Adams at al. which categorizes strokes based on the cause as (1) Large artery atherosclerosis, (2) Cardioembolic, (3) Small artery occlusion, (4) Stroke of Other determined etiology and (5) stroke of undetermined etiology.[6] More specifically:

1. Large artery atherosclerosis:
    a. >50% stenosis or occlusion of ipsilateral intracranial or extracranial major brain artery or branch cortical artery presumably due to atherosclerosis
    b. History of recurrent TIA with same symptoms
    c. Cortical or cerebellar lesions and brainstem or subcortical hemispheric infarcts >1.5cm
    d. Diagnosis cannot be made without vessel imaging or if imaging shows minimal/no changes.
2. Cardioembolism
    a. Stroke due to embolus arising from the heart.
    b. Must have at least one cardiac source identified (**Supplementary Table 3**)
    c. Evidence of previous Transient Ischemic Attack or stroke in more than one vascular territory or another systemic embolism
    d. Must rule out large-artery atherosclerotic sources.
    e. Stroke + medium risk source and no other possible cause = possible cardioembolism
3. Small artery occlusion
    a. <1.5cm lesion on imaging
    b. Subcortical lesion
    c. No evidence of cortical dysfunction (aphasia, neglect)
    d. History of diabetes or hypertension is supportive.
    e. Rule out cardiac sources (**Supplementary Table 3**) or large artery (no stenosis >50% of same-side artery)
4. Stroke of other determined etiology
    a. Less common causes identified through specific investigations (e.g., vasculitis, dissection).
    b. Must exclude all other categories of TOAST.
5. Stroke of undetermined etiology
    a. When no clear cause is identified despite a thorough evaluation, including imaging and relevant investigations.
    b. Excluded: All other categories of TOAST.
    c. Examples:
        i. Atrial fibrillation + ipsilateral stenosis >50%
        ii. Lacunar syndrome + ipsilateral stenosis >50%
        iii. Medium-risk cardiac source + another possible cause of stroke

Patients in which we had limited access to full diagnostic tests and/or discharge summary that could be used to ascertain cause are labeled as such with a “Limited Diagnostic Information” indicator (N=30).

Laboratory/Vital Sign Values were obtained through structured RPDR database. The first value present within eight hours before and up to thirty-six hours after the designated presentation date were used to account for labs that occurred prior to first radiographic scan and/or clinically determined presentation date time. Mean time from presentation to first laboratory value was 3 minutes (0.05 hours) with a standard deviation of 4.08 hours. Reasons for lab values taken prior to presentation time is due to presentation time definition—labs in the emergency department occasionally predated official "presentation time." Vital signs were taken from chart review of the History and Physical.

Glucose was obtained through structured RPDR. Logical Observation Identifiers Names and Codes (**LOINC**) determined by internal review: 2345-7, 2341-6, X1727-7, 6777-7, 2339-0, and XC425-9.

*White Blood Cell Count (**WBC**)* was obtained through structured RPDR. LOINC determined by internal review: 6690-2, 6743-9, 11156-7.

Sodium was obtained through structured RPDR. LOINC determined by internal review: 2951-2, X1737-6, X1015-7, 2947-0.

Creatinine was obtained through structured RPDR. LOINC determined by internal review: 2160-0, X1742-6, X1006-6, X7059-9, X1490-2.

Serum Osmolality was obtained through structured RPDR. LOINC determined by internal review: 2692-2, X1012-4.

Blood Urea Nitrogen was obtained through structured RPDR. LOINC determined by internal review: 3094-0, X1004-1.

*Hemoglobin A1c (**HbA1c**)* was obtained through structured RPDR. Used the HbA1c measurement closest to presentation time. Limited measurements to be ± 90 days from presentation

Blood Pressure (In patients from 2015-2021, available longitudinal blood pressure data during admission was used from the Partners enterprise data warehouse (**EDW**). In patients prior to 2015, all patients underwent chart review to obtain admission blood pressure from clinical notes. In cases in which multiple blood pressures were recorded in a clinical note, the most recent blood pressure from the examination portion of the note was used.

Temperature (In patients from 2015-2021, available longitudinal temperature data during admission was used from the Partners EDW)

Pulse (In patients from 2015-2021, available longitudinal pulse data during admission was used from the Partners EDW)

To remove clearly erroneous lab or vital sign errors due to incorrect chart entry, each longitudinal measurement was assigned a Z-score based on the distribution of the data in the full cohort. Any measurement with an absolute Z-score greater than or equal to three, or 25% change from a preceding value, the observation was flagged as a potential erroneous measurement. The principal investigator examined each patient's flagged laboratory/vital sign trajectories and determined which outlying values fit in the context of their hospital course and which needed to be removed. Temperature that appeared to be in Celsius (30°s- low 40°s) was converted to Fahrenheit. All other abnormally low temperature values were removed (ex: 78°). One sodium value of 102 mEq/L was removed after chart verification. All glucose values ≥1000 mg/dL, and one glucose measurement of 866 mg/dL was removed after chart verification

For **radiographic features**, we used a Natural Language Processing algorithm with high sensitivity described previously[7] to screen for patients with acute MCA stroke identified via ICD-9, ICD-10 codes. All patients with possible large acute MCA stroke were ***visually confirmed*** to have a stroke size ≥ 1/2 of the MCA territory ***using direct visualization*** of radiology images by designated, trained M.D members of the research team (SC, CJO). To be included in 1/2 MCA territory images had to have either full superior or inferior MCA division involvement, or include sufficient volume (estimate of <70cc) of both superior/inferior or deep structures to be included. All indeterminate images underwent secondary review (CJO). A random sampling of our 30 patients using an ABC/2 method[8] demonstrated that median volume was 122 cc^3.

Hemorrhage was recorded by manual inspection according to ECASS II criteria. Petechial Hemorrhage was determined to be HI1 or HI2, and Parenchymal Hemorrhage PI1 or PI2.[9] (1) MRI assessment: *Assessing for petechial and parenchymal hemorrhages on MRI was conducted by utilizing the T2*-weighted gradient-echo (GRE) sequence, with further examination using the Susceptibility Weighted Imaging (SWI) sequence if available. The presence of larger confluent petechial hemorrhage or parenchymal hemorrhage was evident with the use of T2*-GRE whereas SWI, when available, was useful in cases of subtle petechial hemorrhage.* (2) Non-Contrast CT assessment: evaluation for hemorrhage on CT scans was conducted by detecting hyperdensities that could demonstrate hemorrhagic lesions. Each region of interest was evaluated for evidence of hemorrhage, distinguishing between petechial and parenchymal hemorrhage. Percent agreement between trained team members (SC, CJO) on a 10% sample was 92.5%.

*The Alberta Stroke Program Early CT Score (**ASPECTS**)* divides the brain parenchyma into 10 separate non-overlapping regions, three regions at the level just rostral to the ganglionic structures and four at the level of the thalamus.[10] For each baseline scan, each region was deemed affected based on the radiographic evidence of ischemia of the brain parenchyma, hypodensity, loss of cortical ribbon and sulcal effacement on non-contrast CT scan (NCCT) and the restriction of diffusion on Diffusion-MRI (DWI-MRI). For an individual region to count as affected, the lesion should be at least 10% of the respective area. ASPECTS score of 10 implies a totally healthy parenchyma, only at the two levels that the regions are examined, and a score of 0 implies that all the predetermined areas have been affected by ischemia. Percent agreement for ASPECTS areas between two trained M.D. team members (SC, BB) ranged from 72.1-93%.

Midline shift was measured at the level of the septum pellucidum by a trained member of the team using imaging viewer software [Client Outlook, eUnity Diagnostic Viewer, version 6.10.2-489, for MacOs] by navigating to the level of the septum pellucidum at the slice of maximum midline shift. The reviewer created a line connecting the attachment of falx cerebri anteriorly (frontal most notch visible on the scan) and the occipital protuberance (the most posterior notch seen on the posterior aspect of the skull (Image). Windows were set at W:30 L:30. The distance between the midline and the septum pellucidum at both the most lateral and medial boundaries was measured by adding a line perpendicular to the midline. The average of the lateral and medial boundary was used for the final midline shift used in analysis. The results were recorded in a csv file and the difference between the manually recorded MLS and the MLS extracted from the radiology reports were calculated. Mean error was 0.19.

There were no scheduled time points of imaging, however, the median frequency of imaging data within the first 48 and 72 hours was 8.16 [3.97-16.4] and 10.6 [4.98-19.9] hours respectively, suggesting that most patients were closely observed to detect our endpoint. Factors associated with less frequent imaging were age, congestive heart failure, and discharge to home. Factors associated with more frequent imaging were admission sodium, parenchymal HT, ACA vessel occlusion, DHC, and indeterminate death.

Hospital related outcome information was obtained as following.

Death information at discharge or hospice disposition was used.

MLS ≥ 5mm was a dichotomous, outcome variable signaling if at any point in a patient's course of treatment they had a radiographic image in which a midline shift of greater than or equal to 5mm was measured. The date and time of the first image on which this magnitude of shift occurs is also recorded.

Mass effect-related death was obtained via death and/or discharge note review. Investigators determined whether death occurred most likely secondary to mass effect versus other causes. Death due to mass effect was chosen when patients had evidence of increasing mass effect with at least 5mm of MLS up until 120 hours after stroke, although cases were screened up to two weeks on a case-by-case basis to capture possible delayed swelling. Strokes in which patients expired due to prior DNR/DNI and aspiration were not coded as a mass effect-related death. Deaths in which multiple confounding factors including pulmonary edema and/or embolism, sepsis, cancer with poor prognosis, or heart failure were not labeled as a mass effect death.

*Do Not Resuscitate (**DNR**), Do Not Intubate (**DNI**), Comfort Measures Only (**CMO**)* (The date was determined via clinical notes which mentioned any of the following:

- *DNR* (No chest compressions, No defibrillation or electrical cardioversion, No antiarrhythmics, No CPR, No shock).
- *DNI* (No endotracheal intubation, No mechanical ventilator, No non-invasive ventilatory support [CPAP, BiPAP]).
- *CMO* (Comfort only, Comfort measures only, Comfort care, ICM (intensive comfort measure)). The protocol for CMO included removal of bedside monitoring, discontinuation of IV fluids, tube feeds, and all medications with the exception of acetaminophen, morphine, or glycopyrrolate, extubation if appropriate, DNR and DNI orders implemented.

If neither DNR nor DNI was made before CMO, date for CMO was used for both DNR and DNI.

EXPOSURES AND OUTCOMES

Primary Exposure

Hemorrhagic Transformation (**HT**) was defined as a three-factor variable based on the observed hemorrhages from the radiographic images (CTs or Magnetic Resonance (**MR**) Images). Because MR which is more sensitive to blood, was used as well as CTs, we note the heterogeneity in classification for petechial hemorrhage in our limitations section. In all, 825

(98.2%) of patients underwent CT imaging and 649 (77.3%) underwent MR imaging. Only 18 (2.1%) of patients underwent both CTA and MRA imaging.

1. No Hemorrhage: patients with no evidence of bleeding on any of their radiographic scans were classified as having no HT.
2. Petechial Hemorrhage: patients who had evidence of only and exclusively petechial bleeds across all their scans were classified as having petechial HT. The report date time of the scan on which a petechial bleed was first observed was used to approximate the start of the petechial hemorrhagic transformation.
3. Parenchymal Hemorrhage: patients who had evidence of parenchymal bleeds (including those concurrent with petechial HT) in any of their scans were classified as having parenchymal HT. The report date time of the scan on which a parenchymal bleed was first observed was used to approximate the start of the parenchymal hemorrhagic transformation.

ECASS Subtype Breakdown

Grading of petechial and parenchymal HT occurred per the European Co-operative Acute Stroke Study (**ECASS II**) classification scheme of Hemorrhagic Infarction 1 or 2 (**HI-1**, **HI-2**) (petechial) and Parenchymal Hematoma 1 or 2 (**PH-1**, **PH-2**) respectively[11] on head computed tomography (**CT**) or Magnetic Resonance Imaging. Direct visualization of imaging by a trained MD (SC) was used to make ECASS II designations. Percent agreement between trained team members (SC, CJO) on a 10% sample was 92.5%. Of patients with petechial HT, 187 (46.4%) were HI-1 only, and 216 (53.6%) were HI-2. Of patients with parenchymal HT, 36 (45.6%) were PH-1 and 43 (54.4%) were PH-2. Twenty-two parenchymal HT (2.6%) occurred prior to LTME, and 57 (6.8%) occurred prior to DHC.

Outcomes

1. Primary: Life-Threatening Mass Effect (**LTME**)
    a. LTME is defined as a dichotomous outcome based on radiographic images displaying a shift of the midline greater than or equal to 5mm or a history of decompressive surgery.
    b. The time of LTME is defined as either the first instance of an image with a MLS greater than or equal to 5mm or the time of DHC, whichever occurred first.
        i. MLS was determined through verified, automatic processing. The radiology reports associated with each of our patient scans were processed by our natural language algorithm to accurately (0.19 mean error from 10% sampling) extract the degree of midline shift in each image. Any patient with one or more scan with an extracted midline shift of greater than or equal to 5mm was considered to have meet the outcome.
        ii. DHC was determined by a manual review of a patient's chart on EPIC. A trained team member reviewed each patient's medical history to determine whether a patient had decompressive surgery, and if so, what time the surgery occurred.
2. Secondary: Mass effect-related death
    a. Mass effect death is a chart reviewed outcome for patients whose death is explicitly related to mass effect. A member of the team reviewed the charts of all patients who died within 2 weeks and had a midline shift greater than 5mm and

death to confirm if their cause of death was related to their mass effect. This review was done to create an outcome that accounted for the competing risks of death our patient population experienced.

3. Exploratory: All-cause Death or Hospice
    a. The exploratory outcome of all-cause death or hospice was determined based on data from patient H&P and Discharge Summaries from the RPDR. Patients were determined to have this outcome if, for their relevant encounter, they were discharged with a status of "death," "expired," "deceased," or "hospice," by discharge.
4. Exploratory: Disposition
    a. Disposition was defined as discharges to long-term care, hospice, or death versus rehabilitation or home. All patients had an available disposition.
5. Exploratory: DHC
    a. Occurrence of DHC as described above

ANALYSIS

Descriptive Analyses: In the descriptive tables, our team tested the difference in proportions and mean of potential predictors and calculated p-values to evaluate differences across the strata of HT subtype: No HT, Petechial HT, and Parenchymal HT. For dichotomous, nominal, and ordinal variables, a chi-square was used to calculate a p-value for the difference in proportions. For numeric variables, a one-way analysis of variance (**ANOVA**) was used.

Univariate Analyses: To test for confounding relationships, our team created several simple, multinomial logistic regressions predictions HT subtype with the one predictor of interest. In the multinomial logistic regressions, the "No HT" subtype was used as the reference group. The results of these univariate analyses are presented in Supplementary Table 4.

Survival Analysis for Primary Outcome: For time-to-LTME, we used Cox Proportional Hazard Regression and performed Schoenfeld Residuals to test the proportionality in our original models.[12] To effectively study the time-to-LTME stratified by HT subtype, adjusting for covariates, we used an extension of the Cox Proportional Hazards model with time-dependent covariate of HT subtype in our final models. Using our time-dependent exposure approach, HT varies with time and we estimate the HR for the association between HT and the rate of the outcome. We used the R "survival" library with functions Surv and coxph to build our models and structured our data in a counting process structure. Covariates in these analyses were patient age, sex, admission mean arterial pressure, admission glucose, tPA, mechanical thrombectomy, time to mechanical thrombectomy, NIH Stroke Score, prior antiplatelet use, prior anticoagulant use, admission temperature, and MCA stroke side.

When LTME and HT occurred concurrently, we assumed that HT occurred prior to LTME. While it is mechanistically plausible that extreme cerebral edema and midline shift can cause HT, we submit that this inverse causal relation would more likely occur in cases with much larger MLS and herniation.

Final Models for Secondary and Exploratory Outcomes: In our final models, we created three multivariable logistic regression models to test the relationship between parenchymal HT

subtype and our secondary/exploratory outcomes, controlling for confounders observed in prior literature and our univariate analyses (**Supplementary Table 5**).

To account for the observation that cohorts may have differed pre- and post-2015 when thrombectomy use became more wide-spread, we conducted a sensitivity analysis in which we included a “post 2015” dichotomous indicator as a covariate to our model. We defined as any date on or after January 1st, 2015.The findings from this adjusted model were notably consistent with our primary analysis. Further details are presented in Supplementary Table 6.

Missing Values: Supplementary Table 2 displays the number and percent of missing values in our main cohort of patients and all analyzed subgroups. For our analyses, multiple imputation chained equations were used to impute any missing values based on observed values. This was done to avoid excluding any patients from our analyses due to missingness. We used the R package “MICE” to perform these chained imputations. We created 10 imputed datasets, then pooled together the results of the individual analyses on each imputed dataset.

SUPPLEMENTARY TABLES

Supplementary Table 1. Mechanical Thrombectomy Date Time Information (n=142)

**Time-to-Puncture (hours)** (59; 41.5)	**Time-to-Recanalization (hours)** (51; 35.9)	**Time-to-Procedure Note (hours)** (67; 47.1)
4.52 [3.14-7.02]	5.17 [3.88-7.27]	7.58 [6.46-8.93]

Median [25th - 75th] or No. (%)
Date Times used (Time to Puncture (n=59, 42%, Anesthesia Start (n=9; 6.3%), Time-to-Procedure Note (n=67, 47%) Unclear/unknown (n=1; 0.7%))

Supplementary Table 2. Missing Values (n=840)

Variables	Full Cohort	MLS > 0mm	No Early WLST	MT	Age ≤ 60 Years
**Baseline ASPECTS**	1 (0.1)	0 (0)	1 (0.2)	1 (0.7)	0 (0)
**NIHSS**	84 (10.0)	61 (10.1)	76 (11.6)	0 (0)	28 (10.6)
**MT date time**	1 (0.1)	0 (0)	0 (0)	1 (0.1)	0 (0)
**Admission Labs**					
HbA1c (mmol/mol)	184 (21.9)	147 (24.4)	123 (18.8)	36 (25.4)	62 (23.4)
Glucose (per 10mg/dL)	8 (1.0)	6 (1.0)	5 (0.8)	1 (0.7)	4 (1.5)
Low-Density Lipoprotein (mg/dL)	406 (48.3)	296 (49.1)	316 (48.4)	74 (52.1)	128 (48.3)
White Blood Cell Count (K/UL)	9 (1.1)	6 (1.0)	4 (0.6)	1 (0.7)	3 (1.1)
Creatinine (mg/DdL)	9 (1.1)	6 (1.0)	5 (0.8)	2 (1.4)	3 (1.1)
Sodium (mEq/L)	8 (1.0)	6 (1.0)	4 (0.6)	1 (0.7)	3 (1.1)
**Admission Vital Signs**					
Systolic Blood Pressure (mmHg)	16 (1.9)	11 (1.8)	13 (2.0)	5 (3.5)	7 (2.6)
Diastolic Blood Pressure (mmHg)	68 (8.1)	49 (8.2)	54 (8.3)	10 (7.0)	30 (11.3)
Mean Arterial Pressure (mmHg)	68 (8.1)	49 (8.2)	54 (8.3)	10 (7.0)	30 (11.3)
Pulse (beats per minute)	604 (71.9)	419 (69.5)	485 (74.3)	76 (53.5)	191 (72.1)
Temperature (Fahrenheit)	604 (71.9)	419 (69.5)	485 (74.3)	76 (53.5)	191 (72.1)

No. (%)

Abb.: ASPECTS, Alberta Stroke Program Early CT Score; MLS, Midline Shift; MT, Mechanical Thrombectomy; NIHSS, National Institutes of Health Stroke Score; WLST, Withdrawal of Life Sustaining Therapy

Factors with no missing values: Age, Sex, Race, Ethnicity, Hypertension, Atrial Fibrillation, Congestive Heart Failure, Prior Stroke, Antiplatelets, Dual Antiplatelets, Anticoagulant, Warfarin, Direct Oral Anticoagulant, Low Molecular Weight Heparin, Statin, Non-Statin Lipid Lowering Agents, Inpatient Care Prior to Stroke, Presentation to Mass General Brigham <24hrs from Last Seen Well, Stroke Side, Noted Vessel Occlusion, Evidence of ACA infarction, Hemorrhagic Transformation, Tissue Plasminogen Activator, Mechanical Thrombectomy, Midline Shift, Osmotic Therapy, Decompressive Hemicraniectomy, Withdrawal of Life Sustaining Therapy (Do Not Resuscitate, Do Not Intubate, Comfort Measure Only), All-Cause Death or Hospice, Confirmed Mass Effect-Related Death, Life Threatening Mass Effect, Discharge (Hospice, Long Term Care, Rehabilitation, Home

Supplementary Table 3. Features of TOAST Classification of Subtypes of Ischemic Stroke

Features	Subtype			
	Large-artery atherosclerosis	Cardioembolism	Small−artery occlusion (lacune)	Other cause
**Clinical**				
Cortical ort cerebellar dysfunction	+	+	−	+/−
Lacunar syndrome	−	−	+	+/−
**Imaging**				
Cortical, Cerebellar, brain stem or subcortical infarct >1.5 cm	+	+	−	+/−
Subcortical or brain stem infarct <1.5 cm	−	−	+/−	+/−
**Tests**				
Stenosis of extracranial internal carotid artery	+	−	−	−
Cardiac source of emboli	−	+	−	−
Other abnormality on tests	−	−	−	+

TOAST, Trial of Org 10172 in Acute Stroke Treatment.

Supplementary Table 4. Univariate logistic regression by hemorrhage transformation subtypes

n=840	Petechial Hemorrhage		Parenchymal Hemorrhage	
	OR	CI (2.5-97.5)	OR	CI (2.5-97.5)
**Demographics**				
Age	0.99	0.98-1.00	0.99	0.97-1.00
Female	0.69	0.52-0.92	0.46	0.28-0.76
Race		-		-
Black	1.21	0.67-2.19	1.15	0.42-3.18
Asian	1.60	0.72-3.55	2.91	1.02-8.31
Other[a]	1.26	0.82-1.95	1.06	0.49-2.31
Ethnicity		-		-
Hispanic/Latino	1.09	0.45-2.66	0	0- >1000
**Past Medical History**				
Hypertension	0.60	0.44-0.83	0.79	0.46-1.38
Atrial Fibrillation	0.88	0.66-1.17	0.82	0.50-1.34
Congestive Heart Failure	0.79	0.58-1.08	0.56	0.31-1.00
Prior Stroke	0.50	0.32-0.79	0.46	0.19-1.12
**Home Medication**		-		-
Antiplatelets	0.83	0.61-1.14	0.90	0.53-1.54
Dual Antiplatelets	0.46	0.20-1.04	0.52	0.12-2.30
Anticoagulant	1.02	0.65-1.61	1.42	0.71-2.86
Warfarin	1.17	0.71-1.93	1.23	0.54-2.80
Direct Oral Anticoagulants	0.44	0.13-1.47	2.33	0.68-7.94
Low Molecular-Weight Heparin	2.69	0.54-13.4	0	0- >1000
Statin	0.99	0.72-1.35	1.44	0.86-2.39
Non-Statin Lipid Lowering Agents	1.29	0.55-3.06	1.01	0.21-4.75
**Presentation Information**				
Inpatient Care Prior to Stroke	0.38	0.22-0.67	0.20	0.05-0.85
Presented to MGB <24hrs from LSW	0.64	0.44-0.92	0.69	0.38-1.27
Right Sided Stroke	1.25	0.94-1.66	1.42	0.87-2.31
Admission ASPECTS	0.96	0.92-1.01	0.95	0.87-1.04
NIH Stroke Score	1.00	0.98-1.03	1.00	0.96-1.04
Noted Vessel Occlusion	1.06	0.80-1.42	0.83	0.50-1.37
Vessel Occlusion Location		-		-
Internal Carotid Artery	1.06	0.73-1.56	0.79	0.40-1.58
M1	1.01	0.67-1.51	1.00	0.51-1.97
M2	0.99	0.54-1.79	0.55	0.16-1.91
M3 or M4	>1000	0- >1000	0.28	0- >1000
Evidence of ACA infarction	1.13	0.81-1.59	1.45	0.84-2.50
Admission Labs				
HbA1c (per 0.1 mmol/mol)	1.02	1.00-1.03	1.02	0.99-1.04
Glucose (per 10 mg/dL)	1.02	1.00-1.05	1.02	0.98-1.06
Low-Density Lipoprotein (per 10 mg/dL)	1.03	0.98-1.09	1.01	0.92-1.11
White Blood Cell Count (K/UL)	1.00	0.98-1.02	1.00	0.96-1.04
Creatinine (mg/DdL)	0.89	0.73-1.09	1.14	0.90-1.44
Sodium (per 5 mEq/L)	1.14	0.93-1.39	1.48	1.04-2.09
Admission Vital Signs				
Systolic Blood Pressure (per 10 mmHg)	1.00	0.95-1.05	1.01	0.93-1.10
Diastolic Blood Pressure (per 10 mmHg)	1.03	0.94-1.12	1.02	0.87-1.19
Mean Arterial Pressure (per 10 mmHg)	1.01	0.94-1.10	1.01	0.88-1.16
Pulse (per 10 beats per minute)	1.03	0.90-1.18	0.89	0.73-1.10
Temperature (Fahrenheit)	1.28	1.08-1.52	1.44	1.31-1.59
**Acute Intervention**				
Tissue Plasminogen Activator	1.38	1.03-1.86	1.71	1.04-2.79
Mechanical Thrombectomy	1.59	1.06-2.40	3.81	2.17-6.69
Thrombolysis in Cerebral Infarction Score				

TICI 0	0.46	0.15-1.40	0.17	0.03-0.98
TICI 1	1.08	0.21-5.45	0.81	0.11-5.99
TICI 2a	0.76	0.25-2.31	0.44	0.10-1.88
TICI 2b	4.21	1.15-15.4	3.06	0.71-13.1
**Mass Effect**				
Midline Shift				
Midline Shift ≥ 0mm	4.68	3.34-6.57	13.1	5.16-33.0
Midline Shift ≥ 5mm	4.45	3.14-6.32	22.2	11.9-41.2
Maximum Midline Shift (mm)	1.17	1.11-1.25	1.36	1.26-1.46
Treatment				
Osmotic Therapy	2.18	1.59-3.00	4.30	2.59-7.15
Decompressive Hemicraniectomy	3.72	2.03-6.84	17.6	8.79-35.4
**Withdrawal of Life Sustaining Treatment**				
Within 24 hours	0.51	0.36-0.72	0.69	0.38-1.23
Do Not Resuscitate (DNR)	0.52	0.36-0.73	0.70	0.39-1.25
Do Not Intubate (DNI)	0.37	0.25-0.55	0.58	0.30-1.10
Comfort Measure Only (CMO)	0.34	0.19-0.61	0.94	0.44-2.02
During Admission	0.67	0.50-0.90	0.90	0.55-1.47
DNR	0.66	0.50-0.89	0.90	0.55-1.47
DNI	0.63	0.47-0.85	0.84	0.51-1.38
CMO	0.67	0.49-0.90	1.01	0.61-1.67
**Outcomes**				
Death or Hospice by Discharge	0.65	0.48-0.88	1.14	0.69-1.86
Death by Discharge	0.75	0.55-1.03	1.42	0.86-2.35
Confirmed Mass Effect Death	1.89	1.14-3.12	6.16	3.30-11.5
Indeterminate Death	0.38	0.24-0.61	0.20	0.06-0.66
Other Death	0.81	0.46-1.42	0.48	0.14-1.64
Life-Threatening Mass Effect	4.30	3.07-6.03	27.18	13.9-53.3
**Disposition**				
Hospice	0.52	0.29-0.94	0.42	0.12-1.40
Long Term Care	1.00	0.70-1.43	0.93	0.50-1.73
Rehabilitation	1.67	1.24-2.23	1.09	0.66-1.82
Home	0.53	0.25-1.14	0.24	0.03-1.84

Hemorrhage transformation reported includes full 7 days

Abb.: ACA, Anterior Cerebral Artery; ASPECTS, Alberta Stroke Program Early CT Score; CI, Confidence Interval; LSW, Last Seen Well; OR, Odds Ratio

[a]Other Races, includes American Indian, Alaska Native, Native Hawaiian, Pacific Islander, Not Recorded, Not Given or Unknown

Supplementary Table 5. Exploratory Subgroup Multivariable Logistic and Cox Proportional Hazard Regression

	**Life-Threatening Mass Effect**			**Confirmed Mass Effect Related Death**			**All-Cause Death or Hospice**			**Disposition**			**Decompressive Hemicraniectomy**		
	n[a]	*HR (95 CI)*	*p*	*n*	*OR (95 CI)*	*p*	*n*	*OR (95 CI)*	*p*	*n*	*OR (95 CI)*	*p*	*n*	*OR (95 CI)*	*p*
**Subgroup 1:** Midline Shift > 0mm, Total n=603 (71.7%)															
**Parenchymal HT**	45	10.29 (6.75-15.1)	<0.01	74	4.81 (2.30-10.7)	<0.01	74	1.56 (0.78-3.11)	0.21	74	1.59 (0.82-3.05)	0.17	52	2.71 (1.08-6.81)	0.03
**Petechial HT**	322	3.53 (2.72-4.55)	<0.01	339	1.24 (0.71-2.17)	0.44	339	0.78 (0.51-1.19)	0.25	339	0.89 (0.58-1.37)	0.61	339	1.14 (0.66-1.96)	0.65
**Subgroup 2:** No Withdrawal of Life Sustaining Treatment within 24 hours, Total n=653 (77.7%)															
**Parenchymal HT**	34	9.03 (5.61-14.3)	<0.01	62	6.42 (2.68-15.4)	<0.01	62	1.65 (0.75-3.60)	0.21	62	1.76 (0.91-3.40)	0.09	40	4.37 (1.69-11.3)	<0.01
**Petechial HT**	322	3.44 (2.61-4.52)	<0.01	335	1.60 (0.83-3.07)	0.16	335	0.99 (0.64-1.55)	0.97	335	0.95 (0.65-1.40)	0.81	335	1.39 (0.81-2.40)	0.24
**Subgroup 3:** Patients with Mechanical Thrombectomy, n=142 (16.9%)															
**Parenchymal HT**	16	68.7 (30.5-155.1)	<0.01	27	24.0 (3.27-175.5)	<0.01	27	2.18 (0.37-12.7)	0.39	27	3.14 (0.75-13.1)	0.12	19	5.12 (0.81-31.0)	0.08
**Petechial HT**	73	29.1 (15.6-54.3)	<0.01	72	3.01 (0.54-16.8)	0.21	72	0.53 (0.14-2.04)	0.36	72	0.78 (0.26-2.29)	0.65	77	2.02 (0.47-8.74)	0.35
**Subgroup 4:** Age ≤ 60 Years Old, n=265 (31.5%)															
**Parenchymal HT**	11	40.5 (17.2-95.1)	<0.01	30	4.16 (1.00-17.3)	0.05	30	0.89 (0.21-3.81)	0.88	30	2.25 (0.81-6.25)	0.12	13	8.65 (1.91-39.2)	<0.01
**Petechial HT**	129	10.9 (7.28-16.4)	<0.01	135	0.56 (0.19-1.67)	0.30	135	0.44 (0.20-0.95)	0.04	135	0.71 (0.38-1.32)	0.28	133	1.41 (0.74-2.68)	0.30
**Sensitivity Analysis:** HT Subtype defined only by CT Scans, n=798 (95.0%)															
**Parenchymal HT**	41	7.65 (4.85-13.1)	<0.01	76	9.89 (4.77-20.5)	<0.01	76	1.72 (0.92-3.24)	0.09	76	1.71 (0.95-3.10)	0.08	54	4.67 (1.86-11.7)	<0.01
**Petechial HT**	336	2.33 (1.82-2.98)	<0.01	370	2.21 (1.28-3.81)	<0.01	370	0.82 (0.58-1.18)	0.29	370	0.92 (0.65-1.30)	0.62	366	1.50 (0.87-2.58)	0.15

Exploratory Association of Parenchymal HT (primary) and petechial HT (secondary) and outcomes in subgroups. Covariates adjusted for: Age, Sex, Heart Failure, Mean Arterial Pressure, Glucose, Tissue Plasminogen Activator, Mechanical Thrombectomy, Hours to Mechanical Thrombectomy, National Institute of Health Stroke Score, Prior Antiplatelet Use, Prior Anticoagulation Use, Admission Temperature, Right Sided Stroke, Decompressive Hemicraniectomy for all-cause death or hospice and disposition).

Abb.: CI, Confidence Interval; HR, Hazard Ratio; OR, Odds Ratio

[a]Because of different subgroups or outcomes, HT exposure classification differed and number of patients with parenchymal/petechial HT are listed in "n"

Supplementary Table 6. Cohort characteristics pre- and post-2015

Variable	Total n=840	Time Period 2006-2014 n=520 (61.9)	2015-2021 n=320 (38.1)	p-value
**Demographics**				
Age	67.9 (15.5)	67.3 (16)	68.9 (14.7)	0.13
Female	405 (48.2)	252 (48.5)	153 (47.8)	0.91
Race				<0.01
White	649 (77.3)	424 (81.5)	225 (70.3)	
Black	53 (6.3)	28 (5.4)	25 (7.8)	
Asian	33 (3.9)	13 (2.5)	20 (6.2)	
Other[a]	105 (12.5)	55 (10.6)	50 (15.6)	
Ethnicity				1
Hispanic	20 (2.4)	12 (2.3)	8 (2.5)	
**Past Medical History**				
Hypertension	598 (71.2)	361 (69.4)	237 (74.1)	0.17
Atrial Fibrillation	396 (47.1)	250 (48.1)	146 (45.6)	0.54
Heart Failure	248 (29.5)	183 (35.2)	65 (20.3)	<0.01
Prior Stroke	93 (11.1)	69 (13.3)	24 (7.5)	0.01
**Home Medications**				
Antiplatelets	246 (29.3)	181 (34.8)	65 (20.3)	<0.01
Dual Antiplatelets	28 (3.3)	22 (4.2)	6 (1.9)	0.1
Anticoagulants	98 (11.7)	63 (12.1)	35 (10.9)	0.68
Warfarin	77 (9.2)	57 (11)	20 (6.2)	0.03
Direct Oral Anticoagulants	16 (1.9)	4 (0.8)	12 (3.8)	<0.01
Low Molecular-Weight Heparin	8 (1)	5 (1)	3 (0.9)	1
Statins	247 (29.4)	173 (33.3)	74 (23.1)	<0.01
Non-Statin Lipid Lowering Agents	24 (2.9)	19 (3.7)	5 (1.6)	0.12
**Presentation Information**				
LSW to Presentation (hours)	7.3 [3.9 - 17.8]	6.5 [3.8 - 18.4]	8.8 [4.7 - 17.5]	0.11
TOAST Stroke Subtype[b]				0.13
Large Artery Atherosclerosis	96 (11.4)	64 (12.3)	32 (10)	
Cardioembolic	347 (41.3)	204 (39.2)	143 (44.7)	
Small Vessel Occlusion	0 (0.0)	0 (0.0)	0 (0.0)	
Other Known Cause of Stroke	60 (7.1)	45 (8.7)	15 (4.7)	
Unknown Cause of Stroke	307 (36.5)	190 (36.5)	117 (36.6)	
Limited Diagnostic Information	30 (3.6)	17 (3.3)	13 (4.1)	
Right Sided Stroke	435 (51.8)	257 (49.4)	178 (55.6)	0.09
Admission ASPECTS	4.4 (2.9)	4.6 (2.9)	4.1 (2.9)	0.03
NIHSS	17.2 (6.2)	17 (5.9)	17.5 (6.5)	0.31
Vessel Occlusion Location	335 (39.9)	245 (47.1)	90 (28.1)	0.23
Internal Carotid Artery	151 (45.1)	104 (42.4)	47 (52.2)	
M1	129 (38.5)	96 (39.2)	33 (36.7)	
M2	51 (15.2)	41 (16.7)	10 (11.1)	
M3 or M4	4 (1.2)	4 (1.6)	0 (0.0)	
ACA Vessel Occlusion	200 (23.8)	124 (23.8)	76 (23.8)	1
Collateral Score	506 (60.2)	357 (68.7)	149 (46.6)	<0.01
No Collaterals	41 (8.1)	17 (4.8)	24 (16.1)	
≤ 50 but >0 of occluded MCA	220 (43.5)	146 (40.9)	74 (49.7)	
>50 but <100 of occluded MCA	184 (36.4)	141 (39.5)	43 (28.9)	
100 of collateral supply	61 (12.1)	53 (14.8)	8 (5.4)	
**Acute Intervention**				

Tissue Plasminogen Activator	337 (40.1)	214 (41.2)	123 (38.4)	0.48
Mechanical Thrombectomy	142 (16.9)	63 (12.1)	79 (24.7)	<0.01
TICI Score	142 (16.9)	63 (12.1)	79 (24.7)	<0.01
TICI 0	26 (18.3)	19 (30.2)	7 (8.9)	
TICI 1	10 (7)	6 (9.5)	4 (5.1)	
TICI 2a	28 (19.7)	13 (20.6)	15 (19)	
TICI 2b	40 (28.2)	9 (14.3)	31 (39.2)	
TICI 2c or 3	37 (26.1)	15 (23.8)	22 (27.8)	
Indeterminate	1 (0.7)	1 (1.6)	0 (0.0)	
**Admission Labs**				
HbA1c (mmol/mol)	6.2 (1.3)	6.2 (1.2)	6.4 (1.6)	0.09
Glucose (mg/dL)	150.8 (65.2)	146.5 (57.3)	157.8 (75.8)	0.02
White Blood Cell Count (K/UL)	11.8 (7.6)	11.7 (4.9)	12.1 (10.6)	0.5
Creatinine (mg/DdL)	1.1 (0.8)	1.1 (0.9)	1.1 (0.6)	0.27
Sodium (mEq/L)	138.1 (3.6)	137.7 (3.7)	138.7 (3.5)	<0.01
**Admission Vitals**				
Systolic Blood Pressure (mmHg)	149.2 (29.8)	148.3 (29.5)	150.7 (30.4)	0.27
Pulse (beats per minute)[c]	82.7 (21.1)	N/A	82.7 (21.1)	N/A
Temperature (Fahrenheit)[d]	97.9 (1.2)	N/A	97.9 (1.2)	N/A
**Mass Effect**				
Midline Shift > 0 mm	603 (71.8)	361 (69.4)	242 (75.6)	0.06
Midline Shift ≥ 5mm	295 (35.1)	168 (32.3)	127 (39.7)	0.04
Maximum Midline Shift (mm)	6.2 (4.2)	6 (4.1)	6.6 (4.3)	0.11
Osmotic Therapy	282 (33.6)	128 (24.6)	154 (48.1)	<0.01
Decompressive Hemicraniectomy	100 (11.9)	48 (9.2)	52 (16.2)	<0.01
**Withdrawal of Life Sustaining Treatment**				
Within 24 Hours	187 (22.3)	90 (17.3)	97 (30.3)	<0.01
DNR	186 (22.1)	89 (17.1)	97 (30.3)	<0.01
DNI	149 (17.7)	77 (14.8)	72 (22.5)	<0.01
CMO	70 (8.3)	40 (7.7)	30 (9.4)	0.47
During Admission	353 (42)	194 (37.3)	159 (49.7)	<0.01
DNR	352 (41.9)	193 (37.1)	159 (49.7)	<0.01
DNI	328 (39)	180 (34.6)	148 (46.2)	<0.01
CMO	281 (33.5)	159 (30.6)	122 (38.1)	0.03
**Exposures and Outcomes**				
HT Subtype				<0.01
No Hemorrhage	358 (42.6)	241 (46.3)	117 (36.6)	
Petechial	403 (48)	244 (46.9)	159 (49.7)	
Parenchymal	79 (9.4)	35 (6.7)	44 (13.8)	
Time to Life Threatening Mass Effect (hours)	41.7 [27.5 - 63.0]	49.6 [31.6 - 67.0]	34.7 [25.0 - 56.0]	<0.01
Life Threatening Mass Effect	317 (37.7)	178 (34.2)	139 (43.4)	<0.01
Confirmed Mass Effect-Related Death	100 (11.9)	53 (10.2)	47 (14.7)	0.07
All-Cause Death or Hospice	299 (35.6)	167 (32.1)	132 (41.2)	<0.01
**Disposition**				
Hospice	53 (6.3)	22 (4.2)	31 (9.7)	<0.01
Long Term Care	168 (20)	112 (21.5)	56 (17.5)	0.18
Rehabilitation	342 (40.7)	225 (43.3)	117 (36.6)	0.06
Home	30 (3.6)	16 (3.1)	14 (4.4)	0.43

Mean (SD), Median [25th - 75th], or No. (%)

Abb.: ACA, Anterior Cerebral Artery; ASPECTS, Alberta Stroke Program Early CT Score; LSW, Last Seen Well; NIHSS, National Institutes of Health Stroke Score; MGB, Mass General Brigham; TICI, Thrombolysis in Cerebral Infarction; TOAST-Trial of Org 10172 in Acute Stroke Treatment.

[a]Other Races, includes American Indian, Alaska Native, Native Hawaiian, Pacific Islander, Not Recorded, Not Given or Unknown
[b]Toast Classification: Of the 305 patients with stroke of Unknown Etiology, 42 had evidence of cardioembolic and large artery atherosclerosis or other known cause of stroke; Of 62 patients with other known cause of stroke, 24 had carotid dissection.
[c]Pulse data are not available for patient admissions prior to 2015. Thus, those values are blank and no p-value was computed.
[d]Temperature data are not available for patient admissions prior to 2015. Thus, those values are blank and no p-value was computed.

Supplementary Table 7. Additional Baseline Characteristics and Outcomes (n=840)

Variable	Total n=840	Hemorrhage Transformation Subtypes[a]			p
		**No HT** n=358 (42.6)	**Petechial** n=403 (48.0)	**Parenchymal** n=79 (9.4)	
**Demographics**					
Ethnicity					0.34
Hispanic/Latino	20 (2.4)	9 (2.5)	11 (2.7)	0 (0.0)	-
**Home Medication**					
**Antiplatelets**	246 (29.3)	112 (31.3)	111 (27.5)	23 (29.1)	0.53
Dual Antiplatelets	28 (3.3)	17 (4.7)	9 (2.2)	2 (2.5)	0.14
**Anticoagulant**	98 (11.7)	40 (11.2)	46 (11.4)	12 (15.2)	0.59
Warfarin	77 (9.2)	30 (8.4)	39 (9.7)	8 (10.1)	0.79
Direct Oral Anticoagulants	16 (1.9)	8 (2.2)	4 (1.0)	4 (5.1)	0.04
Low Molecular-Weight Heparin	8 (1.0)	2 (0.6)	6 (1.5)	0 (0.0)	0.28
**Non-Statin Lipid Lowering Agents**	24 (2.9)	9 (2.5)	13 (3.2)	2 (2.5)	0.83
**Presentation Information**					
**ACA Vessel Occlusion**	200 (23.8)	79 (22.1)	98 (24.3)	23 (29.1)	0.39
**Collateral Score**	506 (60.2)	219 (61.2)	246 (61.0)	41 (51.9)	0.16
No Collaterals	41 (8.1)	24 (11.0)	16 (6.5)	1 (2.4)	-
≤ 50 but >0 of occluded MCA	220 (43.5)	82 (37.4)	116 (47.2)	22 (53.7)	-
>50 but <100 of occluded MCA	184 (36.4)	86 (39.3)	85 (34.6)	13 (31.7)	-
100 of collateral supply	61 (12.1)	27 (12.3)	29 (11.8)	5 (12.2)	-
**Admission Vital Signs**					
Diastolic Blood Pressure (mmHg)	79.6 (16.5)	79.2 (16.5)	79.9 (16.4)	79.7 (17.4)	0.85
Mean Arterial Pressure (mmHg)	103.1 (19.0)	102.8 (19.0)	103.3 (18.9)	103.2 (19.4)	0.93
**Acute Intervention**					
TICI Score	142 (16.9)	43 (12.0)	72 (17.9)	27 (34.2)	0.06
TICI 0	26 (18.3)	14 (32.6)	10 (13.9)	2 (7.4)	-
TICI 1	10 (7.0)	3 (7.0)	5 (6.9)	2 (7.4)	-
TICI 2a	28 (19.7)	11 (25.6)	13 (18.1)	4 (14.8)	-
TICI 2b	40 (28.2)	4 (9.3)	26 (36.1)	10 (37.0)	-
TICI 2c or 3	37 (26.1)	11 (25.6)	17 (23.6)	9 (33.3)	-
Indeterminant	1 (0.7)	0 (0.0)	1 (1.4)	0 (0.0)	-
**Withdrawal of Life Sustaining Therapy**					
During Admission	353 (42.0)	168 (46.9)	150 (37.2)	35 (44.3)	0.02
DNR	352 (41.9)	168 (46.9)	149 (37.0)	35 (44.3)	0.02
DNI	328 (39.0)	160 (44.7)	136 (33.7)	32 (40.5)	<0.01
CMO	281 (33.5)	135 (37.7)	116 (28.8)	30 (38.0)	0.02
**Dispositions**					
Hospice	53 (6.3)	31 (8.7)	19 (4.7)	3 (3.8)	0.05
Long Term Care	168 (20.0)	72 (20.1)	81 (20.1)	15 (19.0)	0.97
Rehabilitation	342 (40.8)	124 (34.6)	189 (46.9)	29 (36.7)	<0.01
Home	30 (3.6)	18 (5.0)	11 (2.7)	1 (1.3)	0.12

Mean (SD) or No. (%)
Abb.: ACA, Anterior Cerebral Artery; ASPECTS, Alberta Stroke Program Early CT Score; LSW, Last Seen Well; NIHSS, National Institutes of Health Stroke Score; MGB, Mass General Brigham; TICI, Thrombolysis in Cerebral Infarction
[a]Hemorrhage transformation reported includes full 7 days
[b]Other Races, includes American Indian, Alaska Native, Native Hawaiian, Pacific Islander, Not Recorded, Not Given or Unknown

Supplementary Table 8. Hemorrhagic Transformation (HT) reclassification at clinically relevant outcomes (n=840)

Event	HT Subtype[a]	
	**Petechial HT ONLY** n=403 (48.0)	**Parenchymal HT** n=79 (9.4)
Number of patients with HT after LTME	38 (9.4)	29 (36.7)
Number of patients with HT after DHC	16 (4.0)	22 (27.8)

No. (%)
Abb.: DHC, Decompressive Hemicraniectomy; LTME, Life-Threatening Mass Effect
[a]Hemorrhagic transformation reported includes full 7 days

Supplementary Table 9. Image modality frequencies

	**All Imaging Modalities**	**CT or CTA**	**MRI or MRA**
**Image Occurrence**	3617	2968	649
**Number of Patients**	840	825	549
**Occurrence/patient**	4 [3-5]	3 [2 - 5]	1 [1 - 1]
**Frequency**	15.85 [6.6 - 24.93]	19.52 [9.33 - 28.38]	34.3 [18.37 - 68.93]

Abb.: CT, Computed Tomography; CTA, Computed Tomography Angiography; MRI, Magnetic Resonance Imaging; MRA, Magnetic Resonance Angiography

In the first 48 hours after LSW, higher imaging frequency is associated with age, admission sodium, hemorrhagic transformation sub-type, heart failure, anterior circulation artery vascular distribution infarct, DHC, and discharge to home. While these factors do not fully elucidate the exact reasons for close surveillance, we infer that the rational is multifactorial (i.e., system-based common practice in which patients are imaged at 24 hours, in conjunction with an increased concern for malignant edema given the large ischemic stroke cohort.

Supplementary Table 10. Additional time to events by hemorrhage transformation subtypes

Time to Event	Total (n=840)	Hemorrhagic Transformation Subtype[a]		
		No HT (n=358; 42.6%)	Petechial (n=403; 48.0%)	Parenchymal (n=79; 9.4%)
**Time to Acute Treatment**				
Hours to Tissue Plasminogen Activator	2.2 [1.6-2.9]	2.1 [1.6-2.8]	2.2 [1.6-3.0]	2.6 [1.9-3.2]
Hours to Mechanical Thrombectomy	6.5 [4.3-8.8]	7.4 [5.8-8.9]	6.2 [4.4-7.8]	5.3 [3.9-9.1]
**Time to Hemorrhage[b]**				
Hours to Hemorrhage	36.4 [25.1-66.2]	-	39.8 [26.2-72.3]	25.3 [18.8-34.3]
**Time to Mass Effect**				
Hours to 1st Image with MLS	27.7 [18.7-51.1]	26.8 [17.7-43.8]	28.8 [20.7-54.8]	24.9 [15.4-33.1]
Hours to 1st Image with MLS ≥5mm	46.4 [28.1-68.4]	50.9 [32.8-70.0]	50.3 [31.4-74.7]	31.6 [24.9-47.9]
**Time to Mass Effect: Treatment**				
Hours to DHC	43.6 [31.3-61.6]	34.9 [26.9-48.3]	44.6 [31.6-58.0]	40.4 [32.6-63.0]
Days to Osmotic Therapy[c]	1.6 [1.0-2.6]	1.5 [0.8-2.4]	1.9 [1.2-2.7]	1.4 [1.0-2.4]
**Time to WLST**				
Days to Do Not Resuscitate	2 [1-4]	1 [1-3]	2 [1-4.75]	2 [1-6]
Days to Do Not Intubate	2 [1-5]	2 [1-4]	3 [2-6]	3 [1.5-7.5]
Days to Comfort Measures Only	4 [2-7]	3 [2-6]	4 [3-7]	3 [2-8]
**Time to Death**				
Days to Death	6.5 [4-11]	6.5 [4-11]	6 [4-10.5]	7 [3-9]
**Time to Discharge**				
Length of Stay (days)	9 [5-15]	8 [4-13]	9 [6-16]	12 [6-19]

Median [25th - 75th]

Abb.: DHC, Decompressive Hemicraniectomy; HT, Hemorrhage Transformation; MLS, Midline Shift; WLST, Withdrawal of Life Sustaining Treatment

[a]Hemorrhagic transformation classified by any HT within seven days of LSW

[b]Median [25th – 75th] of First Hemorrhage (Petechial or Parenchymal) of Parenchymal Hemorrhage Patients. Many Parenchymal

[c]Osmotic therapy includes mannitol, sodium chloride 3%, and sodium chloride 23

Supplementary Table 11. Time-to-LTME multivariable Cox proportional hazard regression (n=840) with addition post-2015 covariate

**Variables**	**Time to Life-Threatening Mass Effect**	
	HR (95 CI)	P
**Parenchymal Hemorrhage**	8.28 (5.55 – 12.4)	<0.01
**Petechial Hemorrhage**	2.44 (1.89 – 3.15)	<0.01

Abb.: LTME, Life-Threatening Mass Effect; CI, Confidence Interval; HR, Hazard Ratio
Primary exposure: parenchymal HT; Secondary exposure: petechial HT. Adjusted for age, sex, admission mean arterial pressure, admission glucose, tPA, mechanical thrombectomy, time to mechanical thrombectomy, NIH stroke scale, antiplatelet use, anticoagulation use, temperature, stroke laterality, and post-2015

ADDIN EN.REFLIST **REFERENCES**

1. Jensen, P.N., et al., *A systematic review of validated methods for identifying atrial fibrillation using administrative data.* Pharmacoepidemiol Drug Saf, 2012. **21 Suppl 1**(0 1): p. 141-7.
2. Quan, H., et al., *Validation of a case definition to define hypertension using administrative data.* Hypertension, 2009. **54**(6): p. 1423-8.
3. Agarwal, M.A., G.C. Fonarow, and B. Ziaeian, *National Trends in Heart Failure Hospitalizations and Readmissions From 2010 to 2017.* JAMA Cardiol, 2021. **6**(8): p. 952-956.
4. Kokotailo, R.A. and M.D. Hill, *Coding of stroke and stroke risk factors using international classification of diseases, revisions 9 and 10.* Stroke, 2005. **36**(8): p. 1776-81.
5. Kasner, S.E., et al., *Reliability and validity of estimating the NIH stroke scale score from medical records.* Stroke, 1999. **30**(8): p. 1534-7.
6. Adams, H.P., Jr., B. H. Bendixen, L. J. Kappelle, J. Biller, B. B. Love, D. L. Gordon and E. E. Marsh, *Classification of subtype of acute ischemic stroke. Definitions for use in a multicenter clinical trial. TOAST. Trial of Org 10172 in Acute Stroke Treatment.* STROKE.
7. Ong, C., et al., *New Methods of Natural Language Processing using Machine Learning Methods to Identify Ischemic Stroke Presence, Acuity, and Location from Clinical Radiology Reports*, in *Machine Learning For Healthcare*. 2019: Ann Arbor, MI.
8. Sims, J.R., et al., *ABC/2 for rapid clinical estimate of infarct, perfusion, and mismatch volumes.* Neurology, 2009. **72**(24): p. 2104-10.
9. Larrue, V., et al., *Risk factors for severe hemorrhagic transformation in ischemic stroke patients treated with recombinant tissue plasminogen activator: a secondary analysis of the European-Australasian Acute Stroke Study (ECASS II).* Stroke, 2001. **32**(2): p. 438-41.
10. Barber PA, D.A., Zhang J, Buchan AM, *Validity and reliability of a quantitative computed tomography score in predicting outcome of hyperacute stroke before thrombolytic therapy.* Lancet, 2000. **335**(9216): p. 1670-4.
11. Guenego A, L.A., Raymond J, Sabben C, Khoury N, Premat K, Botta D, Boisseau W, Maier B, Ciccio G, Redjem H, Smajda S, Ducroux C, Di Meglio L, Davy V, Olivot JM, Wang A, Duplantier J, Roques M, Krystal S, Koskas P, Collin A, Ben Maacha M, Hamdani M, Zuber K, Blanc R, Piotin M, Fahed R, *Aspiration versus S-Rti. Hemorrhagic transformation after stroke: inter- and intrarater agreement.* Eur J Neurol., 2019.
12. Zhang Z, R.J., Adeleke KA, Pieterse ME, Groothuis-Oudshoorn CGM, *Time-varying covariates and coefficients in Cox regression models.* Ann Transl Med, 2018.
